# Light intensity regulates flower visitation in Neotropical nocturnal bees

**DOI:** 10.1038/s41598-020-72047-x

**Published:** 2020-09-18

**Authors:** Rodolfo Liporoni, Guaraci Duran Cordeiro, Paulo Inácio Prado, Clemens Schlindwein, Eric James Warrant, Isabel Alves-dos-Santos

**Affiliations:** 1 Departamento de Ecologia, Instituto de Biociências, Universidade de São Paulo, Rua do Matão, travessa 14, São Paulo, SP 05508-900 Brazil; 2 Departamento de Botânica, Instituto de Ciências Biológicas, Universidade Federal de Minas Gerais, Caixa Postal 486, Belo Horizonte, MG 31270-901 Brazil; 3 The Lund Vision Group, Department Biology, University of Lund, Sölvegatan 35, 22362 Lund, Sweden

**Keywords:** Plant reproduction, Ecology, Behavioural ecology, Tropical ecology, Zoology, Animal behaviour, Animal physiology, Entomology

## Abstract

The foraging activity of diurnal bees often relies on flower availability, light intensity and temperature. We do not know how nocturnal bees, which fly at night and twilight, cope with these factors, especially as light levels vary considerably from night to day and from night to night due to moon phase and cloud cover. Given that bee apposition compound eyes function at their limits in dim light, we expect a strong dependence of foraging activity on light intensity in nocturnal bees. Besides being limited by minimum light levels to forage, nocturnal bees should also avoid foraging at brighter intensities, which bring increased competition with other bees. We investigated how five factors (light intensity, flower availability, temperature, humidity, and wind) affect flower visitation by Neotropical nocturnal bees in cambuci (*Campomanesia phaea*, Myrtaceae). We counted visits per minute over 30 nights in 33 cambuci trees. Light intensity was the main variable explaining flower visitation of nocturnal bees, which peaked at intermediate light levels occurring 25 min before sunrise. The minimum light intensity threshold to visit flowers was 0.00024 cd/m^2^. Our results highlight the dependence of these nocturnal insects on adequate light levels to explore resources.

## Introduction

Most bees are active during the day, especially under sunny and clear skies^1^. The ability to fly at low light intensities evolved at least 19 times independently. However, approximately 1% of the known bee species—*ca.* 250—are nocturnal, and the obligate dim-light taxa are distributed among the families Andrenidae, Apidae, Colletidae and Halictidae^2^. These species forage in search of flowers in very low light intensities at night, between sunset and sunrise^3,4^. They include crepuscular bees that fly only during dawn and/or dusk, and truly nocturnal bees—such as the Paleotropical Indian carpenter bee *Xylocopa tranquebarica*^5,6^—that also fly during night-time hours, which are defined by the time interval between the two astronomical twilight times at dusk and dawn^2,4^. Moreover, many Neotropical crepuscular bees forage under a dense rainforest canopy that reduces light levels by about 100 times^4^ and may thus experience light intensities similar to those experienced at night in more open habitats. Therefore, nocturnal bees in general seem to be rare and have some relation to light levels in their environments.

According to several authors^1,2,7–10^, when compared with diurnal groups, we still know little about the basic biology of most nocturnal bees, especially concerning their behaviour and interactions with plants. The current knowledge refers mainly to nesting biology^11–15^, abundance and seasonality^16^, nocturnal activity hours^17,18^ and feeding resources^9,15,19^, especially in the genera *Megalopta* (Halictidae) and *Ptiloglossa* (Colletidae). Thus, many questions remain open, such as what environmental factors affect nocturnal bee activity, and especially how this activity is affected by light limitations.

The main factors that influence bee foraging activities are flower availability, temperature and light intensity, while relative air humidity and wind speed often play a secondary role^20^. Host flower abundance can positively affect the number of bees visiting a plant and their flower visitation rate^21^. Moreover, given that bees need to reach a minimum thoracic temperature to fly^22^, and avoid overheating during flight, foraging activity is determined by thermal constraints^23^. Finally, certain minimum light levels seem to be important for triggering first flights^24–26^. The relationships between these factors are complex and which factor is of greatest importance depends on the context^24–27^. At night, temperatures and light levels are lower than during the day, and this could especially affect the behaviour of nocturnal bees^2,28^.

Being able to navigate in dim light seems to be a major challenge faced by bees when foraging at night^4^. Light levels at night are up to 100 million times dimmer than those during the day, and all bees have apposition compound eyes, which are better adapted for brighter environments^29^. Insects that adopted a nocturnal lifestyle early in evolutionary history, such as most moths and many beetles, evolved superposition compound eyes whose optical design improves light capture by between two and three orders of magnitude compared to apposition eyes^4,30,31^. In a typical apposition compound eye, such as found in bees, each ommatidium catches light from a given direction in space exclusively through its own small corneal lens (usually just a few tens of micrometres in diameter) and this tiny pupil limits the lowest light intensity for which vision remains reliable^4,30^. Thus, as light levels decrease after sunset, the apposition compound eyes of bees operate at their physiological limits^4,30,32^. Therefore, for nocturnal bees, their relatively recent evolutionary transition from a diurnal to a nocturnal lifestyle represented an invasion into a new and extreme environment^32^, where light intensity likely plays a major role in their foraging behaviour.

Nocturnal bees are likely to be more affected by light levels than by other environmental factors, not only because of the extremely low intensities of light at night, but also because of its immense daily variation, especially during twilight periods. During dusk or dawn, light intensity varies dramatically, since sun elevation changes very quickly, increasing light levels by at least one million times from night to day^33,34^. Moreover, twilight light levels can also change according to moon phase, moon elevation, cloud cover, and time in the season^33,34^. Thus, bee vision needs to cope with all this variation as well and foraging should be favoured in some, but not all, illuminations. Nonetheless, foraging during twilight and at night, despite these challenges, does have its advantages. Firstly, there is less competition, since most foraging bees are day-active^9,35^. Secondly, many plants bloom only at dawn or at night as a water-saving adaptation^28^. Thirdly, early morning bee foragers may be highly rewarded from unvisited flowers that have accumulated nectar throughout the entire night^36^. Thus, nocturnal bees could be favoured by foraging as early each day as possible. Considering the limitations in their ability to see at the dimmest nocturnal light levels, and their intense competition with a massive number of diurnal bees that start to arrive at flowers around sunrise when light levels are bright enough, we should expect a higher activity of nocturnal bees during the mid-twilight period, prior to the arrival of diurnal bees, when light intensities are at intermediate levels. During this period, nocturnal bees can still access flower resources earlier than other bees, but without compromising their visual capacities (as would have been the case at the dimmest nocturnal light levels). Therefore, during the dawn, foraging activity in nocturnal bees might increase as light levels rise, peak at intermediate twilight intensities, and fall as light levels continue to rise towards sunrise.

**Figure 1:**
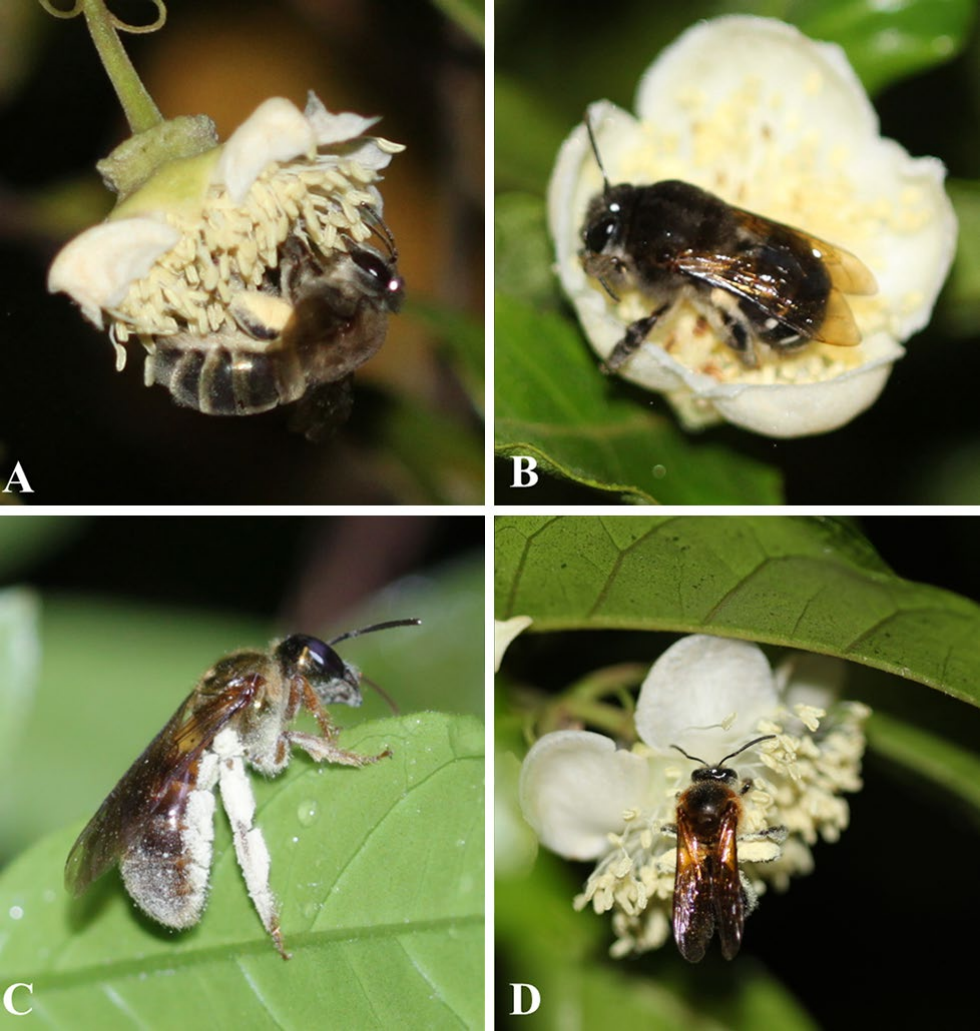
Nocturnal bee species that visit flowers of cambuci (*Campomanesia phaea*, Myrtaceae) in south-eastern Brazil. (**A**) *Ptiloglossa latecalcarata* (Colletidae), (**B**) *Ptiloglossa* sp. (Colletidae), (**C**) *Megalopta sodalis* (Halictidae), (**D**) *Megommation insigne* (Halictidae).

Nocturnal bees can pollinate *Campomanesia phaea* (O. Berg) Landrum (Myrtaceae), a tree species endemic to the Atlantic Forest of south-eastern Brazil and commonly known as cambuci. This plant reaches a height of 5 m, blooms between October and January, and produces an acidic edible fruit^37^. Cambuci flowers are white, open at night, and release a sweet scent. Cambuci trees are mainly pollinated by nocturnal bees^38^ (*Megalopta sodalis* Vachal 1904, *Megommation insigne* Smith 1853, *Ptiloglosssa latecalcarata* Moure 1945, *Ptiloglossa* sp., and *Zikanapis seabrai* Moure 1953), which makes it an excellent study system for our investigation (Fig. 1).

Here we investigate how light intensity affects the foraging activity of nocturnal bees on cambuci flowers. We also measure other environmental factors that could interfere with this relationship, such as flower availability, temperature, humidity and wind speed. Our hypotheses are: (1) light intensity is the main environmental factor that influences foraging activity in nocturnal bees, and (2) nocturnal bees are more active at intermediate light intensities in the twilight period. We also expect that foraging activity can start earlier on brighter nights, such as moonlit nights. Our findings demonstrate that the interaction between nocturnal bees and flowers strongly depends on light intensity. We discuss how this dependence can be explained by ecological requirements and evolutionary processes.

## Results

We observed flowers for 7,614 min (or 127 h) over 30 days during morning twilight. The beginning of the observation period varied from 5:12 h during the first twilight (17 October 2017) to 4:45 h during the last twilight (24 November 2017), while the end of the observation period varied from 6:59 h during the first twilight to 6:39 h during the last twilight. This disparity reflects the variation in day length throughout the season, which resulted in an increase of 7 min in the total observation period from the first (108 min) to the last (115 min) twilight period.

### Environmental factors

Light intensity levels varied in a predictable way during each twilight period, following a sigmoid curve. These levels were mainly controlled by sun elevation relative to the horizon (Fig. S1). Other environmental variables did not vary considerably within each twilight period but presented different average values for different twilight periods. Average air temperature ranged from 7.6 °C during the coldest twilight to 18.8 °C during the warmest twilight (Figs. S2, S3). All twilight periods were very humid and with little wind, with average relative air humidity ranging from 89 to 100% (Figs. S4, S5) and maximum wind speed varying from 0 to 10.3 km/h (Fig. S6). Orchard flower abundance increased as the flowering season advanced, from 109 to 640 flowers per day (Fig. S7). Therefore, light intensity, air temperature and flower abundance varied considerably among twilight periods, while relative air humidity and wind speed were less variable.

### Visitation rate

The average number of visits per flower per minute for all twilight periods, and for the five species of nocturnal bees taken together, was 2.13 ± 6.46 × 10^–2^ (mean ± SD), which were distributed in 33 cambuci trees (Table 1). *Ptiloglossa latecalcarata*, the most abundant species, had an average of 1.90 ± 6.12 × 10^–2^ (mean ± SD) visits per flower per minute—approximately 90% of the total.

**Table 1:** Visitation rate (mean ± SD) of five nocturnal bee species foraging in a commercial cambuci (*Campomanesia phaea*, Myrtaceae) orchard surrounded by secondary Atlantic forest fragments in Mogi das Cruzes, São Paulo State, Brazil.

Bee family	Bee species		Visitation rate (× 10^–2^ visits/flower/min)
Colletidae	*Ptiloglossa latecalcarata*		1.90 ± 6.12
	*Ptiloglossa* sp.		0.03 ± 0.66
	*Zikanapis seabrai*		0.002 ± 0.15
Halictidae	*Megalopta sodalis*		0.03 ± 0.51
	*Megommation insigne*		0.17 ± 1.39
	Total		2.13 ± 6.46

The visitation rate, on average, did not increase as the cambuci flowering season advanced and flower abundance increased (r^2^ = 0.03; y = 0.0164 + 0.0003x; p = 0.17; Fig. S8), mainly due to the relatively constant visitation rate of the most common bee *P. latecalcarata* (Fig. 1). Other nocturnal bee species had lower frequencies, with two species (*Ptiloglossa* sp. and *Z. seabrai*) recorded only in the last eight sampled twilight periods (Fig. S9).

**Figure 2:**
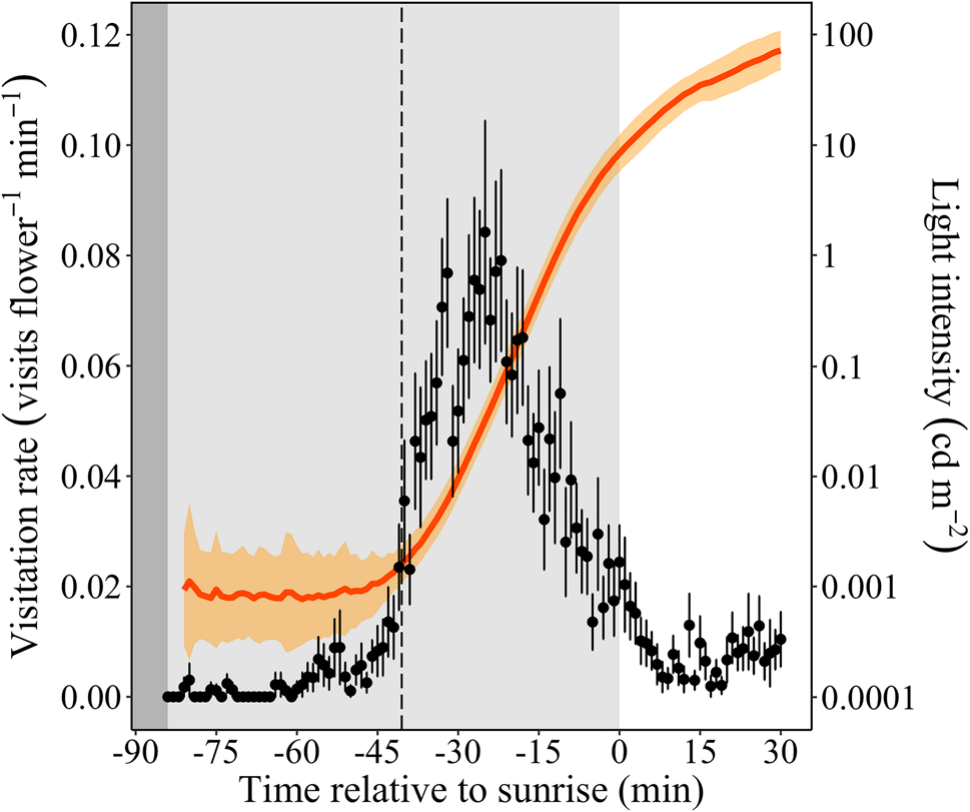
Visitation rate (visits/flower/min) by nocturnal bees on cambuci flowers and light intensity variation during twilight and for 30 min after sunrise. *Black dots* are average visitation rate values and *black bars* are standard errors of the mean (n = 30 twilight periods). The *coloured curve* represents mean light intensity for each minute during twilight and the *shaded area* the relative errors (n = 30 twilight periods). Background colours represent true night (*dark grey*), twilight (*light grey*) and daytime (*white*). The *vertical dashed line* approximately marks mid-twilight. Sunrise time (0 in the time axis) varied between 6:29 h on the first sampled twilight (17 Oct 2017) to 6:09 h on the last twilight (24 Nov 2017).

During twilight, the visitation rate increased and reached a peak 25 min before sunrise, then decreased until 30 min after sunrise, when visits ceased. The visitation rate started to increase substantially during mid-twilight, when ambient light levels also started to increase (Figs. 2, S10). Most nocturnal bee species stopped foraging around sunrise, with the exception of *P. latecalcarata*, which was recorded until 30 min after sunrise (Figs. S11, S12). Diurnal bees, mostly worker bees of *Apis mellifera* (Apidae) and stingless bees (Meliponini), initiated flower visits around 30 min before sunrise (Figs. S13, S14).

### Light intensity is the main factor affecting flower visitation rate of nocturnal bees in cambuci

The main environmental factor explaining cambuci flower visitation rate by nocturnal bees was light intensity. The model accounting for the effects of light intensity (with a quadratic term for light) was the best supported by the data, and no alternative model had an equivalent support (that is ∆AICc < 2, Table 2), with the following equation for standardized variables: Visitation rate = – 2.94 + 0.44 × (light intensity) – 1.60 × (light intensity)^2^ – 0.05 × (temperature) + 0.06 × (humidity) – 0.07 × (wind) + 0.08 × (flowers).

**Table 2:** Model selection for factors affecting foraging activity of nocturnal bees, using generalized linear mixed models (GLMMs) with Poisson distribution.

Models	logLikelihood	AICc	∆AICc	DF	Weight
Light + light^2^ + all other variables	− 3,645.5	7,309.1	0.0	9	1
Light + all other variables	− 4,379.6	8,775.1	1,466.1	8	< 0.001
All variables, except light	− 4,381.0	8,775.9	1,466.9	7	< 0.001
No fixed effects (null)	− 4,670.4	9,342.7	2033.6	1	< 0.001

*AICc* corrected Akaike information criterion, *DF* degrees of freedom.

The response variable was the number of visits per minute, using the number of observed flowers as model offset. The best model includes all environmental variables (light intensity, air temperature, relative air humidity, maximum wind speed, orchard flower abundance) with a quadratic term for light intensity.

Model coefficients indicated that light intensity has a strong effect, while all other environmental variables exerted weak effects (Table S1). Light intensity had a standardized coefficient at least five times larger than the coefficient for the second most important variable (flower abundance). The 95% confidence intervals for estimated coefficients for temperature, relative air humidity, maximum wind speed, and orchard flower abundance included zero (Table S2). Therefore, only light intensity had a considerable and relevant effect on visitation rate.

**Figure 3:**
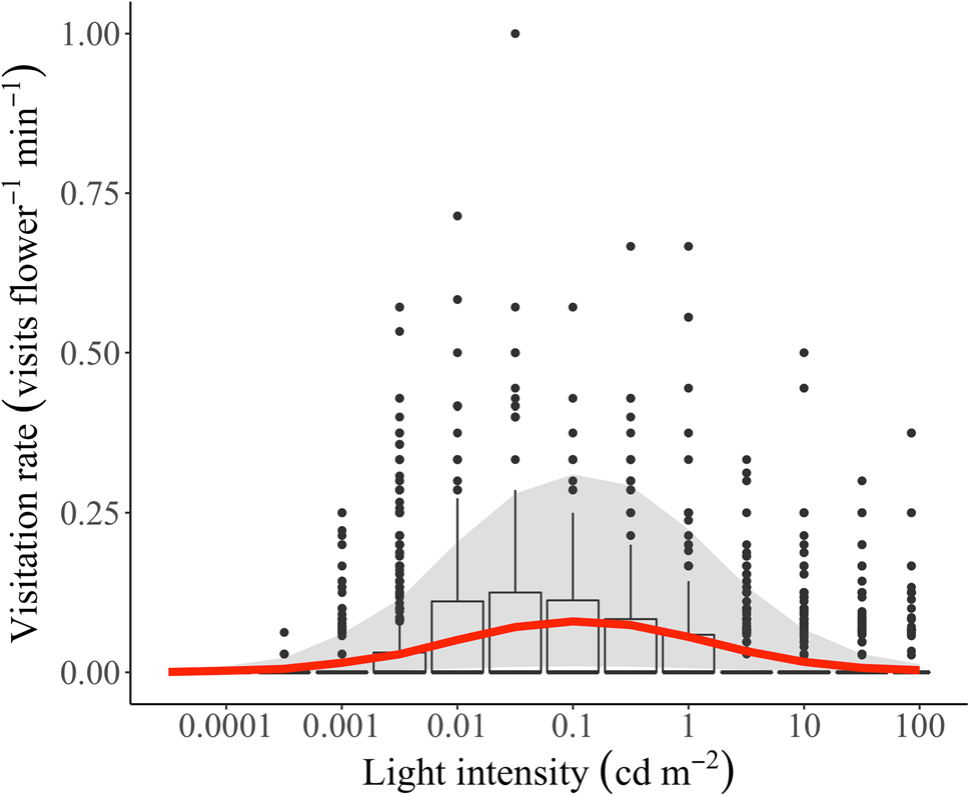
Distribution of visitation rate (visits/flower/min) by nocturnal bees on cambuci flowers during twilight as a function of light intensity measured as luminance (cd/m^2^). *Boxes* delimit where 50% of the values for each light interval are concentrated, *horizontal lines* indicate the median (all of them are zero), and *dots* represent outliers. The *red curve* represents the predicted visitation rate by the best supported model, which includes a quadratic term for the light effect. The 95% confidence interval of predicted values is shown in *light grey*.

We also found that visitation rate peaks at intermediate light intensity levels (Fig. 3), as we expected. According to the best supported model, at a medium light intensity of 0.01 cd/m^2^ nocturnal bees would make 32 times more visits per flower per minute than at the lowest light level of 0.0001 cd/m^2^ and 24 times more visits per flower per minute than at the highest light level of 100 cd/m^2^. The lowest light level that nocturnal bees began to forage in cambuci flowers was recorded as 0.00024 cd/m^2^ (or 2.4 × 10^–4^ cd/m^2^).

**Figure 4:**
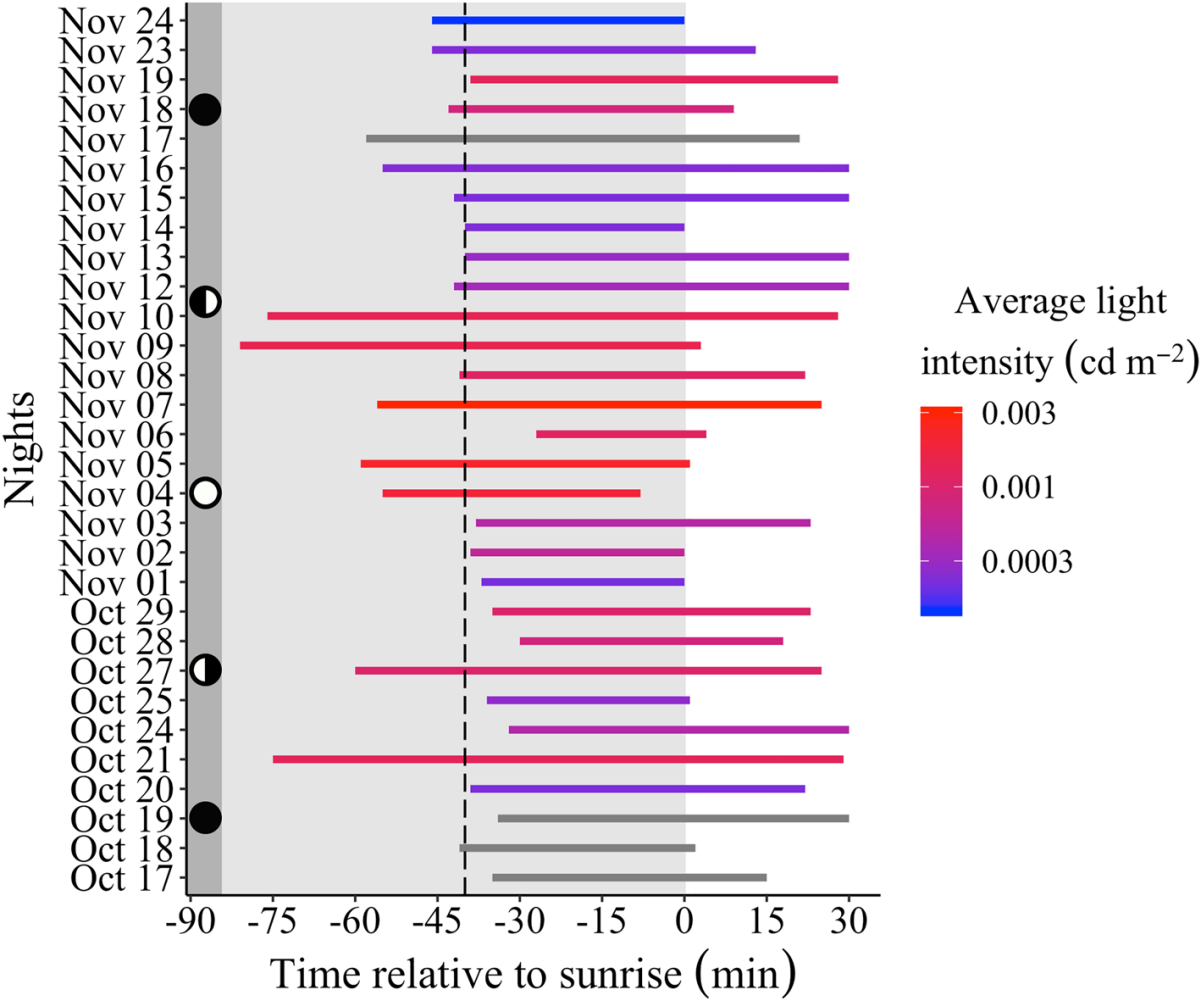
The activity durations of nocturnal bees foraging on cambuci flowers measured during twilight periods over five weeks. Each *horizontal line* represents the time interval during which bees visited cambuci during each twilight period. *Line colours* indicate average light intensity—measured as the average luminance of an 18% grey card (cd/m^2^) during the first half of the twilight when light levels varied considerably between twilight periods (see this variation in Fig. S1). *Grey horizontal lines* are nights with missing light intensity data from the first half of the twilight. *Circles* at left represent moon phases (new moon = *black circle*; full moon = *white circle*). Other plot conventions as in Fig. 2.

From the selected model, we also noted that brighter nights, i.e., those with higher light levels during the first half of the twilight (Fig. S1), permit earlier visits. The earliest measured light intensity levels on brighter nights (e.g. those with a full moon), which were around 0.003 cd/m^2^, were responsible for this increased visitation activity. On darker nights the earliest measured light intensity levels were much dimmer—around 0.0003 cd/m^2^ (see the response of visitation rate to these light intensity levels in the red curve in Fig. 3). Indeed, nights with earlier bee activity seem to be brighter nights (Fig. 4), if we take into account the average light intensity during the first half of the twilight, when light levels varied substantially among twilight periods (Fig. S1).

## Discussion

The foraging activity of nocturnal bees in cambuci flowers during morning twilight can be predicted mainly by light intensity, and other putative environmental factors do not contribute as much to these predictions. The maximum activity occurs at intermediate light intensity levels (around 0.1 cd/m^2^), which usually occur around 30 min before sunrise. The onset of activity occurs at a minimum light intensity threshold of 0.00024 cd/m^2^, which is one of the first recorded thresholds for flower foraging in nocturnal bees (see below). For twilight periods with higher initial light intensity levels, such as during clear moonlit nights, nocturnal bees can start to forage earlier. This light-dependent foraging activity is likely related to a combination of factors, such as visual challenges imposed by dim light levels^31^ and ecological demands to access resources before the onset of visits made by massive numbers of diurnal bees^35^.

It makes sense that light intensity controls nocturnal bee activity because dim light environments represent an extreme situation for bees, which are equipped with apposition compound eyes that are better adapted for brighter conditions^4,32^. We thus expected that the visual capacities of nocturnal bees—and thus their flight and foraging activities—require a minimum light level. Indeed, this threshold exists for other nocturnal species. According to a previous study^39^, a light intensity of at least 0.0001 cd/m^2^—about half of the threshold we found here—is required for foraging activity in *Megalopta genalis* (Halictidae), a bee inhabiting rainforests, while for *Lasioglossum (Sphecodogastra) lusoria* (Halictidae), a bee inhabiting deserts, a light level of at least 0.02 cd/m^2^ is required. In the present study, we provide a minimum light intensity that nocturnal bees require to find flowers in an agro-forest context, where shadows from taller neighbouring trees reduce light levels even more. Our threshold intensity value thus agrees well with that previously reported for *M. genalis* when flying in forests^39^*,* indicating that nocturnal bees foraging on cambuci likely have a similar light intensity threshold. The registered minimum threshold of 0.00024 cd/m^2^ is, surprisingly, approximately 100 times dimmer than the value registered for the desert bee *L. lusoria* foraging in Onagraceae plants^39^. This finding confirms our previous understanding concerning the dimmest conditions in which nocturnal bees can find flowers in general, especially in darker environments such as forests. Thus, our results demonstrate that foraging activity is limited by light intensity and can occur in extremely dim conditions. Moreover, our study confirms the importance of measuring actual light levels in situ rather than using arbitrary descriptors such as “nocturnal”, “crepuscular” or “diurnal” when comparing the behaviours of bees that are dependent on light intensity.

Our results also show that light intensity influences the activity of nocturnal bees in a non-linear manner, contrasting with what we know for diurnal bees^1,40^. Many studies have demonstrated how light intensity affects the onset of foraging activity in diurnal bees^24,25,41,42^. It is generally stated that light controls bee activity in a linear way (based on simple linear regressions)^40^, and consequently, cloudy days should delay the onset of bee activity, and this has been confirmed for several species of solitary and social bees^24–26^. However, this is not the case for the nocturnal bees studied here. These bees still have a strong light-dependent activity, but bee activity peaks at intermediate light levels and then falls as light levels increase even more.

This difference in light-dependent activity between nocturnal bees and diurnal bees begs explanation. Why should higher light intensities following sunrise actually decrease the activity of nocturnal bees? We propose two non-exclusive explanations: one physiological and one ecological. First, nocturnal bees probably have an endogenous clock that sets the exact times to start and to stop foraging, as already demonstrated for other bee species^43,44^. By using environmental cues such as light intensity to entrain their circadian rhythm^45^, bees could predict the best time to cease collecting resources and to start investing in activities within the nest. Second, higher light levels trigger (or coincide with) the arrival of diurnal bees in cambuci flowers, possibly stimulating nocturnal bees to stop their foraging activity to avoid competition. During evolution, nocturnal bees might have changed their preferred foraging period from daytime to night-time to avoid competition and to inhabit a niche relatively free of enemies^2^. This hypothesis was tested for two Neotropical nocturnal bee species in Panama—*Megalopta genalis* and *M. ecuadoria* (Halictidae)—and it was found that nest parasitism rates for these species were four times lower than those of closely related diurnal species^9^. Moreover, there is evidence that interference competition with diurnal bees causes *Megalopta* to cease foraging activity in the morning^35^. We have also observed that diurnal bees, mainly *Apis mellifera*, arrive in large numbers just before sunrise and occupy all the space in cambuci flowers (Figs. S13, S14), which likely deters the activity of nocturnal bees. Therefore, our results support the idea that higher light intensities can be used as a proxy for the end of an activity regulated by a circadian clock and/or an increased competition with diurnal bees, since higher light levels do not prevent nocturnal bees from continuing to see their surroundings (Eric Warrant, personal observation). Indeed, nocturnal bees cease visiting cambuci flowers at light levels of around 100 cd/m^2^, which occurs about 30 min after sunrise, the time which coincides with many diurnal species, especially social bees, on cambuci flowers^38^ (Figs. S11, S12).

Another interesting result we obtained is that light intensity is the main (and perhaps the only) factor controlling the activity of nocturnal bees. Considering that diurnal bees also frequently respond to temperature and flower availability^24,27^, why did flower availability not affect substantially visitation rates in our nocturnal bees? As previously stated, a nocturnal lifestyle brings some advantages for accessing resources prior to potential diurnal competitors^9^, so variations in flower availability might not affect the activity of nocturnal bees as much as it would for the considerably more numerous diurnal bees. Moreover, in our agro-forest context, bees were foraging within a high density of cambuci trees—their apparent main source of pollen—over a relatively small area. This represents a highly artificial condition, since cambuci trees are very likely to be sparser in their natural habitats. For these two main reasons, floral resources were probably not limiting for these bees.

Finally, why did temperature not considerably affect the activities of nocturnal bees on cambuci flowers? As far as we know, only four studies^46–49^ have evaluated the effects of temperature on the activities of nocturnal bees. Two of these studies^46,49^ found no effect of temperature, while one study suggests that temperature positively affects bee activity, but only above 25 °C and combined with lower light intensity^47^. The fourth study found that higher temperatures have a moderately negative effect on activity^48^. Thus, on its own temperature seems to have only a weak to moderate effect on nocturnal bee activity. Moreover, three of the studies also suggest light intensity as a factor affecting bee activity^46–48^. Our study appears to be the first to evaluate how several factors simultaneously affect nocturnal bee activity on a fine scale (i.e. over intervals of one minute) and to determine the exact relation, in terms of magnitude and direction, between environmental variables and the foraging activity of nocturnal bees. Hence, it seems that light intensity affects the activity of nocturnal bees in general, but that the effect of temperature is secondary and varies according to context.

As temperature is relatively constant in tropical environments^28^, light could be the only determining factor for foraging times in Neotropical nocturnal bees. Even though our nocturnal bees did not experience constant temperature—which varied from approximately 8 to 19 °C—bees were able to visit flowers even on the coldest night. This suggests that these low temperatures are not an obstacle for foraging and that perhaps these bees might thermoregulate. Many desert bees have a bimodal activity pattern similar to nocturnal bees and are notably able to overcome low temperatures early in the morning and to avoid overheating in the hottest hours of the day and are thus classified as endothermic animals^50^. For instance, the desert bee *Ptiloglossa arizonensis* can fly very early on cold days and, once in the air, this bee can keep higher thoracic temperatures than its surroundings^46^. This probably explains why the foraging behaviour of our bee *Ptiloglossa latecalcarata*, which is closely related to *P. arizonensis* and likely to have the same thermoregulatory capacities, depends very little on temperature.

Besides using vision, nocturnal bees can also use other sensory information to find flowers in the dark, such as floral volatiles^51,52^. In cambuci flowers, 2-phenylethanol and 1-octanol are the two main compounds, which are preferentially emitted at night, soon after anthesis^38^. However, bees still depend heavily on visual cues to learn the characteristics of the first flowers they encounter and to land on them^53^. Relative to the dark green background of cambuci leaves, cambuci flowers are incredibly bright, with a broad reflection spectrum that likely creates a high contrast white target for a nocturnal bee (Fig. S15). Combining these previous results on scent-mediated flower visitation^38,51,52^ with our findings of a minimum light intensity threshold that permits flower search behaviour (and reflection spectra indicating that cambuci flowers have likely evolved to be highly salient visual targets at night), we suggest that nocturnal bees use both vision and olfaction to find flowers. Diurnal bees combine visual and olfactory cues to recognize and find flowers^54,55^, and our work indicates that this is likely the case for nocturnal bees too. Moreover, these two floral sensory cues seem to be correlated in a community context and when integrated together could enhance bee attraction and pollination^56^. Thus, it would be worthwhile to investigate how nocturnal bees specifically respond to various floral visual cues and how they integrate information from different sensory modalities (e.g. vision and olfaction) during foraging, especially in a community-wide perspective.

In conclusion, light intensity is the main environmental factor affecting the foraging activity of nocturnal bees on cambuci flowers. Their activity during the morning twilight peaks at intermediate light intensity levels, implying that brighter nights, with higher light levels in the first half of the twilight, permit earlier foraging activity. These findings highlight the environmental factors that are relevant for explaining the interaction between nocturnal bees and their host plants. Our results also show that the light-dependent activity of bees is not always linear (as found in many diurnal species). In the case of nocturnal bees, this non-linear activity might be explained in terms of evolutionary advantages to forage earlier, associated with an endogenous clock that possibly sets the time to stop foraging and/or prevents competition from diurnal bees at higher light levels. Intriguing questions that remain for further study include how the different senses used by bees to find flowers (such as olfaction) interact with this light-dependent activity, and how bees, including diurnal species, are affected by ambient light intensity in other contexts.

## Methods

### Study area

Field work was conducted on private farmland (Sítio Cambuci Nativa, 23° 25′ S, 46° 10′ W, 670 m a.s.l.) in Mogi das Cruzes, São Paulo State, Brazil (Fig. S16). The 15-ha farm is located within the Atlantic Forest domain and includes cambuci tree orchards (6 ha) and secondary forest fragments (9 ha). According to an updated Köppen-Geiger classification, the regional climate is type Cfa, humid subtropical, with a hot summer and average annual rainfall over 1,400 mm^57^.

### Study system

Cambuci trees exhibit steady-state flowering, producing few new flowers per day. The hermaphroditic disc flowers measure around three centimetres in diameter, have white petals (Fig. S15) and up to 500 stamens, and offer only pollen as reward^38^. Flowers last only one day and open during the night, between 4:00 and 4:30 h. They are visited by a large diversity of insects, such as wasps, flies and bees, including diurnal and nocturnal bees^38^. The insects visit the flowers at different times of the day: nocturnal bees initiate visits soon after the beginning of anthesis and leave the flowers at sunrise or some minutes later, being followed by diurnal floral visitors until noon, when most of the pollen has already been depleted. However, nocturnal bees are the most effective pollinators^38^.

In the study area, five nocturnal bee species were sampled visiting the flowers of *C. phaea*: *Megalopta sodalis* (Halictidae), *Megommation insigne* (Halictidae), *Ptiloglosssa latecalcarata* (Colletidae), *Ptiloglossa* sp. (Colletidae), and *Zikanapis seabrai* (Colletidae) (Fig. 1; Table 1). Bees of *Megalopta* nest in dead wood^14^, while the other species nest in the soil^15,58^. Colletid species are solitary, while the halictid species are facultatively social^1,14^.

### Flower visitation rate

We counted flower visits per minute to measure bee foraging activity during the morning twilight. Also called dawn in its final part, this period begins at astronomical twilight when the sun is 18° below the horizon and starts to contribute to light levels in the atmosphere (ending true night) and finishes when the sun rises^33^. We also recorded flower visits per minute from sunrise until 30 min after sunrise (when sun elevation is around 6° above the horizon and light intensity reaches a stable value), since nocturnal bees can extend their activity some minutes after sunrise on some days. We counted visits over 30 days, from October to November 2017, covering half of the cambuci flowering season. As twilight duration increases as the summer solstice approaches^59^, the total observation time per twilight period ranged from 108 to 115 min. We did not sample on rainy mornings, since rain limits bee activity and imposes technical limitations on measuring light levels.

During each night, before twilight and anthesis, two or three observers randomly selected one tree each, totalling two or three flowering cambuci trees per night. When the selected tree was not flowering, we moved along the planting line from east to west until a flowering individual was found. Each observer selected a number of flowers to watch during each twilight (average of 12 observed flowers per tree, varying from 4 to 37, according to the number of flowers in the field of view) and stayed close to the tree waiting for bees with their head lantern turned off. When one bee was heard, we turned lanterns on for a few seconds to register the bee species. We recorded, according to the local time, the exact minute of each visit and the bee species observed. Later, we organized raw data as the number of visits for different bee species each single minute. We calculated the flower visitation rate (number of visits per observed flower per minute) as our response variable and discriminated between each tree and each night. We also counted visits from diurnal bee species since they started to arrive when nocturnal bees were still active (Supplementary Information).

### Measurements of environmental factors

Light intensity was measured as luminance (cd/m^2^) of light reflected from an 18% grey card using a very sensitive (measurement range: 0.00001–19.90 cd/m^2^) photometer (ERP-105, Hagner, Sweden) coupled to a data logger (OM-CP-VOLT101A, Omega, Brazil; sample rate: 0.2 Hz). Average light intensity per minute was recorded from the data logger and light data were measured for the entire observation period. The photometer was placed in an open area 10 m away from the orchard border to avoid interference from observers' lanterns (Fig. S16).

Other abiotic factors that could potentially affect bee activity were also measured to control their effects and to better discriminate the effect due to light intensity only. Air temperature and relative air humidity were measured each night with a thermohygrometer (HOBO-U23-001, Onset, USA) installed at flower level in one of the observed cambuci trees. We also measured these climate variables with a meteorological station (H21-USB, Onset, USA) installed in an open area 70–100 m away from the orchards (Fig. S16). We measured maximum wind speed for the entire observation period with a portable cup anemometer (PCE-A420, PCE Instruments, Germany) installed close to the photometer. All devices were equipped with data loggers, and visitation data could be compared with environmental factors minute by minute. We also qualitatively estimated cloud cover in the sky; twenty nights had completely overcast skies while ten were clear starlit nights. Weather conditions were relatively constant throughout the duration of each observed twilight.

Flower availability was estimated as flower abundance in two ways: (1) the total number of recently open flowers in 20 randomly selected cambuci trees each night (orchard flower abundance); and (2) the total number of recently opened flowers in the selected trees (tree flower abundance). We randomly selected 20 different individual trees from which to count flowers after the end of the observation period and repeated the selection each night to detect daily floral resource variation. We counted all recently opened flowers in a tree following branches from bottom to top.

### Data analyses

To evaluate how light intensity affects the activity of nocturnal bees when controlling for other environmental factors, we modelled visit count data using generalized linear mixed models (GLMMs) with a Poisson distribution, and included the number of observed flowers per tree as an offset. All models included the additive effects of environmental factors (light intensity, temperature, humidity, wind speed, and flower abundance) as predictor variables (fixed effects) and an identifier of the tree and the sampling night as possible random effects to account for shared variance of nested sampling units.

Prior to modelling, we standardized all candidate fixed-effect variables and excluded those that were highly correlated (*rho* > 0.5). Thus, we kept only temperature and humidity variables from the meteorological station and only orchard flower abundance estimates, excluding temperature and humidity data from thermohygrometer and tree flower abundance. All analyses were implemented in the R environment^60^ using the following extra packages: *bbmle*^61^*, DHARMa*^62^*, dplyr*^63^*, ggplot2*^64^*, lattice*^65^*, latticeExtra*^66^*, lme4*^67^*, oce*^68^*,* and *tidyr*^69^.

We followed a widely used two-step model selection protocol^70^, starting with a full model, with all fixed effects, but alternative combinations of the random effects, to determine the random-effect structure that is best supported by the data. We selected models using corrected Akaike Information Criteria (AICc). We then kept the random-effect structure from the best supported model and fitted the following models to identify the best supported fixed-effect structure: (1) all environmental variables, except light intensity; (2) all environmental variables, including a linear effect for light intensity; (3) all environmental variables, including a quadratic effect for light intensity; (4) no fixed effects (null model). We included a model with quadratic effect for light intensity to model a scenario where intermediate light intensities produce larger visitation rates, as stated in our second hypothesis. Then, we used the AICc to again identify which relation between light intensity and bee activity (if any) was best supported by the data. We calculated 95% confidence intervals for the estimated fixed effect of each predictor variable in the best model to evaluate how strong the effect of light intensity was compared with other factors. Our hypotheses predicted a quadratic relationship of visitation rate and light intensity, with a peak of activity at intermediate light intensities during twilight. We also expected that the effect of light intensity would be larger than the effects estimated for the other environmental predictors.

We checked if the best model fulfilled the main assumptions of GLMMs using scalar (quantile) residuals created by a simulation-based approach implemented in the package *DHARMa*^62^. We checked the uniformity of scaled residuals and plotted residuals against explanatory variables. We also checked zero-inflation (Fig. S17). Validation occurred after the model selection and before its interpretation. We also checked if foraging activity increased throughout the season with a linear regression between date and the average visitation rate for all minutes and trees on each night (Fig. S8).

## Supplementary information

Supplementary Information.

## Data Availability

All data generated or analysed during this study are included in this published article (and its Supplementary Information files).

## References

[1] Michener CD (2007). The Bees of the World.

[2] Wcislo WT, Tierney SM (2009). Behavioural environments and niche construction: The evolution of dim-light foraging in bees. Biol. Rev..

[3] Warrant EJ (2007). Nocturnal bees. Curr. Biol..

[4] Warrant EJ (2008). Seeing in the dark: Vision and visual behaviour in nocturnal bees and wasps. J. Exp. Biol..

[5] Somanathan H, Borges RM, Warrant EJ, Kelber A (2008). Visual ecology of Indian carpenter bees I: Light intensities and flight activity. J. Comp. Physiol. A Neuroethol. Sensory Neural Behav. Physiol..

[6] Somanathan H, Saryan P, Balamurali GS (2019). Foraging strategies and physiological adaptations in large carpenter bees. J. Comp. Physiol. A Neuroethol. Sensory Neural Behav. Physiol..

[7] Engel MS (2000). Classification of the bee tribe Augochlorini (Hymenoptera: Halictidae). Bull. Am. Museum Nat. Hist..

[8] Silveira FA, Melo GAR, Almeida EAB (2002). Abelhas Brasileiras: Sistemática e Identificação.

[9] Wcislo WT (2004). The evolution of nocturnal behaviour in sweat bees, *Megalopta genalis* and *M. ecuadoria* (Hymenoptera: Halictidae): An escape from competitors and enemies?. Biol. J. Linn. Soc..

[10] Carvalho AT, Maia ACD, Ojima PY, dos Santos AA, Schlindwein C (2012). Nocturnal bees are attracted by widespread floral scents. J. Chem. Ecol..

[11] Janzen D (1968). Notes on nesting and foraging behavior of *Megalopta* (Hymenoptera: Halictidae) in Costa Rica. J. Kansas Entomol. Soc..

[12] Roberts RB (1971). Biology of the crepuscular bee *Ptiloglossa guinnae* N. sp. with notes on associated bees, mites, and yeasts. J. Kansas Entomol. Soc..

[13] Rozen JG (1984). Nesting biology of Diphaglossine bees (Hymenoptera, Colletidae). Am. Museum Novit..

[14] Santos LM, Tierney SM, Wcislo WT (2010). Nest descriptions of *Megalopta aegis* (Vachal) and *M. guimaraesi* Santos & Silveira (Hymenoptera, Halictidae) from the Brazilian Cerrado. Rev. Bras. Entomol..

[15] Sarzetti L, Genise J, Sanchez MV, Farina J, Molina A (2013). Nesting behavior and ecological preferences of five Diphaglossinae species (Hymenoptera, Apoidea, Colletidae) from Argentina and Chile. J. Hymenopt. Res..

[16] Wolda H, Roubik DW (1986). Nocturnal bee abundance and seasonal bee activity in a Panamanian forest. Ecology.

[17] Linsley EG, Cazier MA (1970). Some competitive relationships among matinal and late afternoon foraging activities of Caupolicanine Bees in Southeastern Arizona (Hymenoptera, Colletidae). J. Kansas Entomol. Soc..

[18] Roulston TH (1997). Hourly capture of two species of *Megalopta* (Hymenoptera: Apoidea; Halictidae) at black lights in Panama with notes on nocturnal foraging by bees. J. Kansas Entomol. Soc..

[19] Smith AR, López Quintero IJ, Moreno Patiño JE, Roubik DW, Wcislo WT (2012). Pollen use by *Megalopta* sweat bees in relation to resource availability in a tropical forest. Ecol. Entomol..

[20] Dafni A, Kevan PG, Husband BC (2005). Practical Pollination Biology.

[21] Somanathan H, Borges RM (2001). Nocturnal pollination by the carpenter bee *Xylocopa tenuiscapa* (Apidae) and the effect of floral display on fruit set of *Heterophragma quadriloculare* (Bignoniaceae) in India. Biotropica.

[22] Contrera FAL, Nieh JC (2007). The effect of ambient temperature on forager sound production and thoracic temperature in the stingless bee, *Melipona panamica*. Behav. Ecol. Sociobiol..

[23] Willmer PG (1983). Thermal constraints on activity patterns in nectar-feeding insects. Ecol. Entomol..

[24] Linsley EG (1958). The ecology of solitary bee. Hilgardia.

[25] Figueiredo-Mecca G, Bego LR, Nascimento FS (2013). Foraging behavior of *Scaptotrigona depilis* (Hymenoptera, Apidae, Meliponini) and its relationship with temporal and abiotic factors. Sociobiology.

[26] Streinzer M, Huber W, Spaethe J (2016). Body size limits dim-light foraging activity in stingless bees (Apidae: Meliponini). J. Comp. Physiol. A.

[27] Linsley EG (1978). Temporal patterns of flower visitation by solitary bees, with particular reference to the southwestern United States. J. Kansas Entomol. Soc..

[28] Borges RM, Somanathan H, Kelber A (2016). Patterns and processes in nocturnal and crepuscular pollination services. Q. Rev. Biol..

[29] Warrant EJ (1999). Seeing better at night: Life style, eye design and the optimum strategy of spatial and temporal summation. Vis. Res..

[30] Warrant EJ (2004). Nocturnal vision and landmark orientation in a tropical halictid bee. Curr. Biol..

[31] Warrant E (2004). Vision in the dimmest habitats on Earth. J. Comp. Physiol. A Neuroethol. Sensory Neural Behav. Physiol..

[32] Warrant E, Dacke M (2011). Vision and visual navigation in nocturnal insects. Annu. Rev. Entomol..

[33] Rozenberg GV (1966). Twilight.

[34] O’Carroll DC, Warrant EJ (2017). Vision in dim light: Highlights and challenges. Philos. Trans. R. Soc. B Biol. Sci..

[35] Smith AR, Kitchen SM, Toney RM, Ziegler C (2017). Is nocturnal foraging in a tropical bee an escape from interference competition?. J. Insect Sci..

[36] Kapustjanskij A, Streinzer M, Paulus HF, Spaethe J (2007). Bigger is better: implications of body size for flight ability under different light conditions and the evolution of alloethism in bumblebees. Funct. Ecol..

[37] Lorenzi H (2002). Brazilian Trees: A Guide to the Identification and Cultivation of Brazilian Native Trees.

[38] Cordeiro GD, Pinheiro M, Dötterl S, Alves-dos-Santos I (2017). Pollination of *Campomanesia phaea* (Myrtaceae) by night-active bees: A new nocturnal pollination system mediated by floral scent. Plant Biol..

[39] Kelber A (2006). Light intensity limits foraging activity in nocturnal and crepuscular bees. Behav. Ecol..

[40] Polatto LP, Chaud-Netto J, Alves-Junior VV (2014). Influence of abiotic factors and floral resource availability on daily foraging activity of bees. J. Insect Behav..

[41] Willis DS, Kevan PG (1995). Foraging dynamics of *Peponapis pruinosa* (Hymenoptera: Anthophoridae) on pumpkin (*Cucurbita pepo*) in Southern Ontario. Can. Entomol..

[42] Wcislo WT, Cane JH (1996). Floral resource utilization by solitary bees (Hymenoptera: Apoidea) and exploitation of their stored foods by natural enemies. Annu. Rev. Entomol..

[43] Bellusci S, Marques MD (2001). Circadian activity rhythm of the foragers of a eusocial bee (Scaptotrigona aff depilis, Hymenoptera, Apidae, Meliponinae) outside the nest. Biol. Rhythm Res..

[44] Bloch G, Bar-Shai N, Cytter Y, Green R (2017). Time is honey: Circadian clocks of bees and flowers and how their interactions may influence ecological communities. Philos. Trans. R. Soc. B Biol. Sci..

[45] Enright JT (1970). Ecological aspects of endogenous rhythmicity. Annu. Rev. Ecol. Evol. Syst..

[46] Shelly TE, Villalobos EM, Buchmann SL, Cane JH (1993). Temporal patterns of floral visitation for two bee species foraging on *Solanum*. J. Kansas Entomol. Soc..

[47] Gottlieb D, Keasar T, Shmida A, Motro U (2005). Possible foraging benefits of bimodal daily activity in *Proxylocopa olivieri* (Lepeletier) (Hymenoptera: Anthophoridae). Environ. Entomol..

[48] Franco EL, Gimenes M (2011). Pollination of *Cambessedesia wurdackii* in Brazilian campo rupestre vegetation, with special reference to crepuscular bees. J. Insect Sci..

[49] dos Oliveira FS, Ribeiro MHM, Nunez CV, de Albuquerque MC (2016). Flowering phenology of *Mouriri guianensis* (Melastomataceae) and its interaction with the crepuscular bee *Megalopta amoena* (Halictidae) in the restinga of Lençóis Maranhenses National Park, Brazil. Acta Amaz..

[50] Willmer P, Stone G (1997). Temperature and water relations in desert bees. J. Therm. Biol..

[51] Krug C (2018). Nocturnal bee pollinators are attracted to guarana flowers by their scents. Front. Plant Sci..

[52] Siqueira E (2018). Pollination of *Machaerium opacum* (Fabaceae) by nocturnal and diurnal bees. Arthropod. Plant. Interact..

[53] Orbán LL, Plowright CMS (2014). Getting to the start line: How bumblebees and honeybees are visually guided towards their first floral contact. Insectes Soc..

[54] Burger H, Dotterl S, Ayasse M (2010). Host-plant finding and recognition by visual and olfactory floral cues in an oligolectic bee. Funct. Ecol..

[55] Milet-Pinheiro P, Ayasse M, Schlindwein C, Dobson HEM, Dötterl S (2012). Host location by visual and olfactory floral cues in an oligolectic bee: Innate and learned behavior. Behav. Ecol..

[56] Kantsa A (2017). Community-wide integration of floral colour and scent in a Mediterranean scrubland. Nat. Ecol. Evol..

[57] Peel MC, Finlayson BL, McMahon TA (2007). Updated world map of the Koppen-Geiger climate classification. Hydrol. Earth Syst. Sci..

[58] Michener CD, Lange RB (1958). Observations on the behavior of Brasilian halictid bees, III. Univ. Kansas Sci. Bull..

[59] Meinel AB, Meinel MP (1991). Sunsets, Twilights, and Evening Skies.

[60] R Core Team, R. R: A language and environment for statistical computing. *R Found. Stat. Comput. Vienna, Austria*. www.R-project.org (2017). Accessed 15 Dec 2017.

[61] Bolker, B. & R Core Team, R. bbmle: Tools for general maximum likelihood estimation. *R Packag. version 1.0.20*. https://CRAN.R-project.org/package=bbmle (2017). Accessed 15 Dec 2017.

[62] Hartig, F. DHARMa: Residual diagnostics for hierarchical (multi-level/mixed) regression models. *R Packag. version 0.1.5*. https://CRAN.R-project.org/package=DHARMa (2017). Accessed 15 Dec 2017.

[63] Wickham, H., Francois, R., Henry, L. & Müller, K. dplyr: A grammar of data manipulation. *R Packag. version 0.7.4. *https://CRAN.R-project.org/package=dplyr (2017). Accessed 15 Dec 2017.

[64] Wickham H (2009). ggplot2: Elegant Graphics for Data Analysis.

[65] Sarkar, D. Lattice: Multivariate data visualization with R. *R Packag. version 0.20–38. *https://CRAN.R-project.org/package=lattice (2008). Accessed 15 Dec 2017.

[66] Sarkar, D. & Andrews, F. latticeExtra: Extra graphical utilities based on lattice. *R Packag. version 0.6-28.* https://CRAN.R-project.org/package=latticeExtra (2016). Accessed 15 Dec 2017.

[67] Bates D, Maechler M, Bolker B, Walker S (2015). Fitting linear mixed-effects models using lme4. J. Stat. Softw..

[68] Kelley, D. & Richards, C. oce: Analysis of oceanographic data. *R Packag. version 0.9-22. *https://CRAN.R-project.org/package=oce (2017). Accessed 15 Dec 2017.

[69] Wickham, H. & Henry, L. tidyr: Easily tidy data with ‘spread()’ and ‘gather()’ functions. *R Packag. version 0.8.0*. https://CRAN.R-project.org/package=tidyr (2018). Accessed 15 Dec 2017.

[70] Zuur AF, Ieno EN, Walker NJ, Saveliev AA, Smith GM (2009). Mixed Effects Models and Extensions in Ecology with R.

