# Supplementary material for: Light intensity regulates flower visitation in Neotropical nocturnal bees
Source: Sci Rep. 2020 Sep 18;10:15333. doi: 10.1038/s41598-020-72047-x (PMC7501267; doi:10.1038/s41598-020-72047-x)
Supplement: Supplementary file 1 — Supplementary Information. [file 41598_2020_72047_MOESM1_ESM.zip › 3.NEW_Supplementary_Information_Liporoni et al._srep/3.1.Supplementary_Tables_and_Figures.pdf]

Supplementary Information

Light intensity regulates flower visitation in Neotropical nocturnal bees

Rodolfo Liporoni[1], Guaraci Duran Cordeiro[1], Paulo Inácio Prado[1], Clemens Schlindwein[2], Eric James Warrant[3], and Isabel Alves-dos-Santos[1]

[1] Departamento de Ecologia, Instituto de Biociências, Universidade de São Paulo. Rua do Matão, travessa 14, 05508-900, São Paulo, SP, Brazil.

[2] Departamento de Botânica, Instituto de Ciências Biológicas, Universidade Federal de Minas Gerais. Caixa Postal 486, 31270-901, Belo Horizonte, MG, Brazil.

[3] The Lund Vision Group, Department Biology, University of Lund. Sölvegatan 35, S-22362, Lund, Sweden.

Supplementary Table S1. Summary statistics of the best generalized linear mixed model that includes all variables and a quadratic term for light intensity.

Variable	Estimate	Standard Error
Intercept	-2.938	0.177
Light	0.442	0.038
$Light^2$	-1.602	0.048
Temperature	-0.055	0.142
Humidity	0.064	0.120
Wind speed	-0.069	0.114
Flower abundance	0.078	0.121

Supplementary Table S2. The 95% confidence intervals for the estimates of each predictor variable (fixed effects) of the best generalized linear mixed model. Intervals for tree and night (random effects) are for their standard deviations, which were estimated as 0.71 and 0.60, respectively.

Variables	2.5 %	97.5 %
Intercept	-3.303	-2.589
Light	0.368	0.518
$Light^2$	-1.698	-1.509
Temperature	-0.335	0.240
Humidity	-0.156	0.324
Wind speed	-0.304	0.159
Flower abundance	-0.172	0.320
Tree	0.512	1.011
Night	0.440	0.859

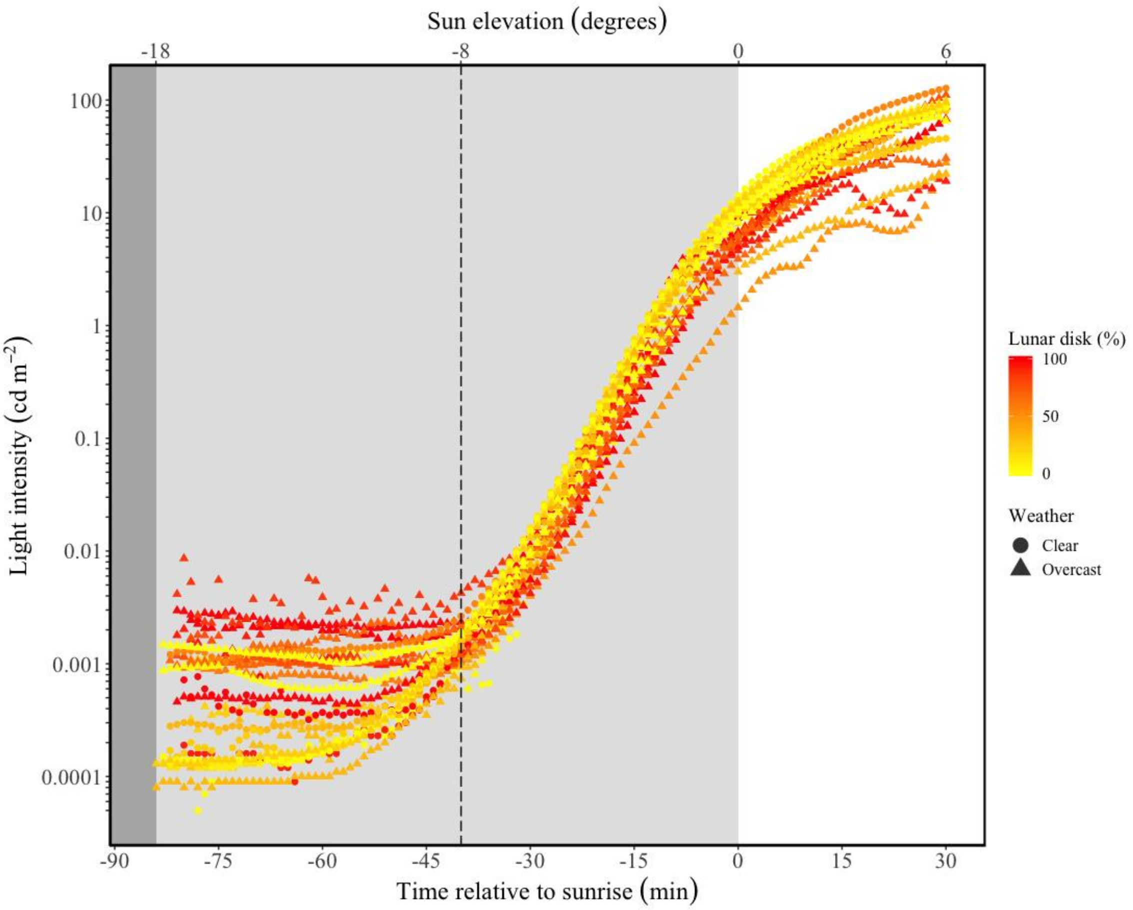

Supplementary Figure S1. Light intensity measured as luminance from an 18% grey card (cd/m^2) as a function of time relative to sunrise (bottom axis) and sun elevation (in degrees) relative to the horizon (top axis) over a number of twilight periods (*sigmoidal curves*). Different curves indicate the visible percentage of the illuminated lunar disk (full moon = 100%, new moon = 0%) and the weather conditions (clear sky or overcast sky). Background colours represent night (*dark grey*), twilight (*light grey*) and daytime (*white*). The *vertical dashed line* approximately marks mid-twilight. Sunrise time (0 on the time axis) varied between 6:29 h during the first sampled twilight (17 Oct 2017) to 6:09 h during the last sampled twilight (24 Nov 2017). Light intensity mainly depends on sun elevation relative to the horizon. However, when sun elevation is lower than -8°, *i.e.*, during the first half of the twilight, the contribution of sunlight to natural illumination is still low but moon phase and/or weather conditions can significantly affect light intensity. Thus, we found variations of approximately two orders of magnitude in the light levels during the first half of the twilight among sampled twilight periods, *i.e.*, until approximately 40 minutes before sunrise (or when sun elevation is lower than -8°). This variation can be explained by a combination of moon phase, moon elevation and weather conditions, since a full moon can increase light levels during the night and the beginning of the twilight by almost two orders of magnitude compared with a clear starlit night, while cloud cover can decrease light levels by one order of magnitude. A full moon is brighter but was lower in the sky during the observed period. So a half-moon during twilight can make a larger contribution to light intensity during the first half of the twilight than a full moon. After this initial variation, light levels become more similar between twilight periods because the increasing sun elevation begins to dominate the natural illumination.

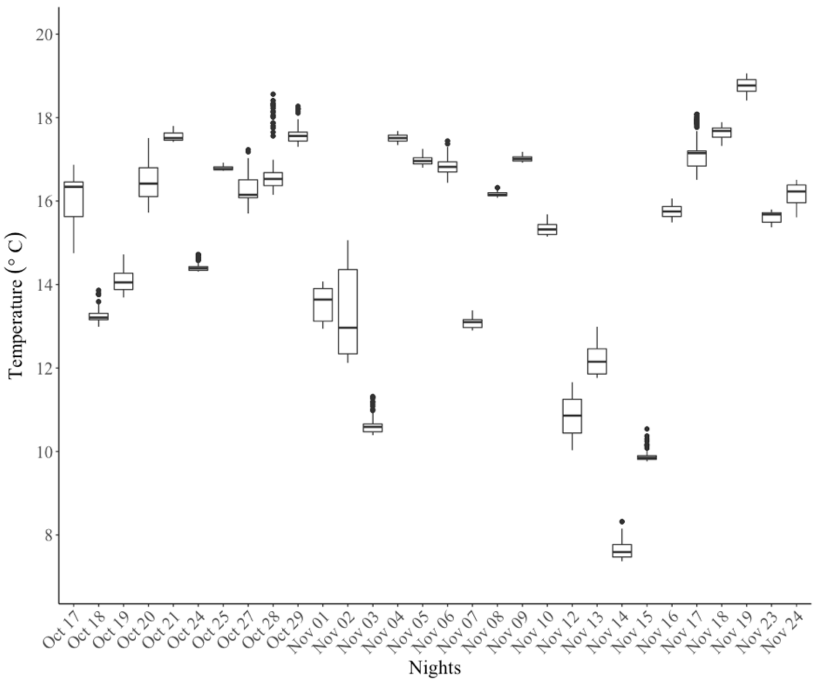

Supplementary Figure S2. The distribution of mean ambient temperatures (°C) measured during the different twilight periods. *Boxes* delimit where 50% of the values are concentrated, *horizontal lines* indicate the median, and *dots* represent outliers.

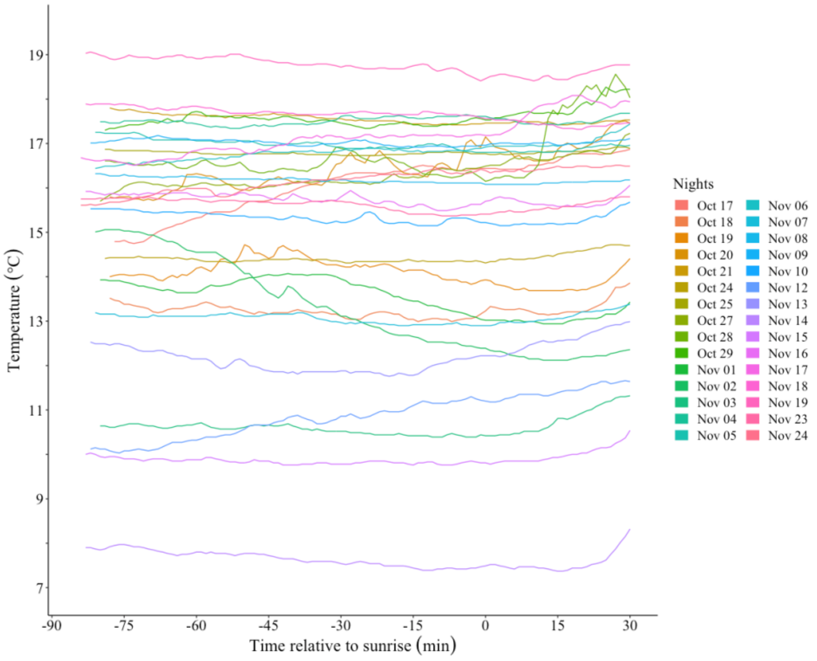

Supplementary Figure S3. The temperature variation (°C) during each sampled twilight period. Each *coloured curve* represents a single sampled twilight. Time axis conventions as in Fig. S1.

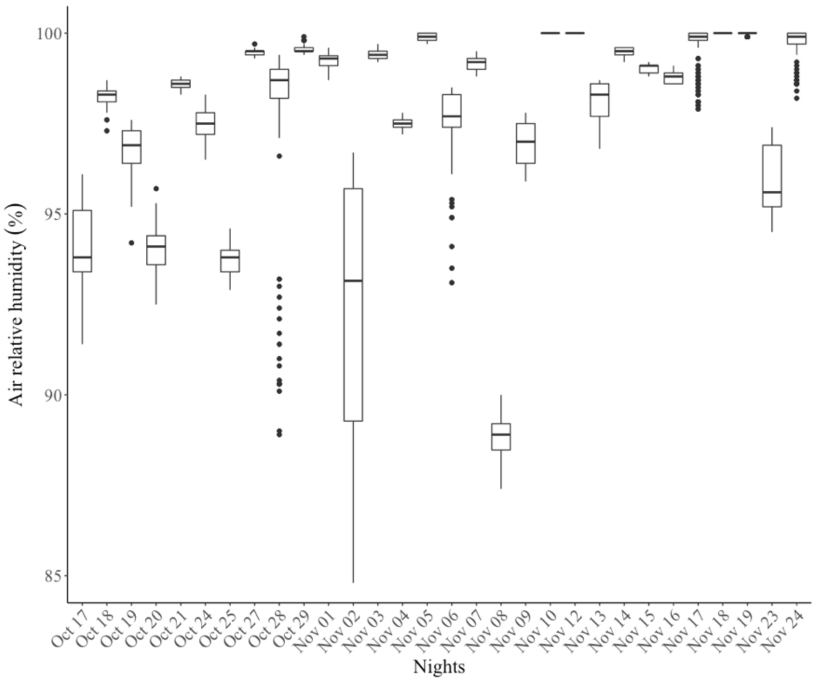

Supplementary Figure S4. The distribution of mean air relative humidity (%) measured during the different twilight periods. Plot conventions as in Fig. S2.

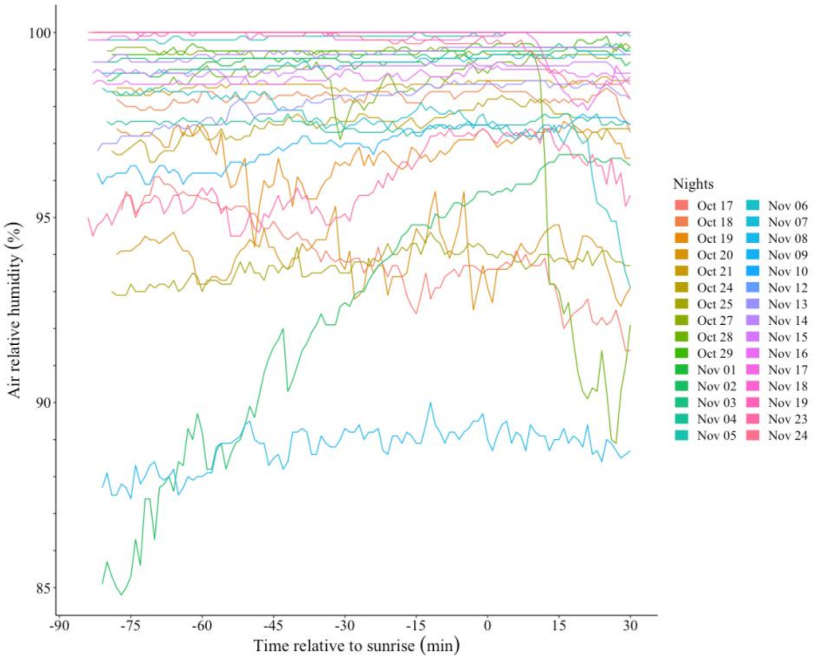

Supplementary Figure S5. The variation in air relative humidity (%) during each sampled twilight period. Each *coloured curve* represents a single sampled twilight. Time axis conventions as in Fig. S1.

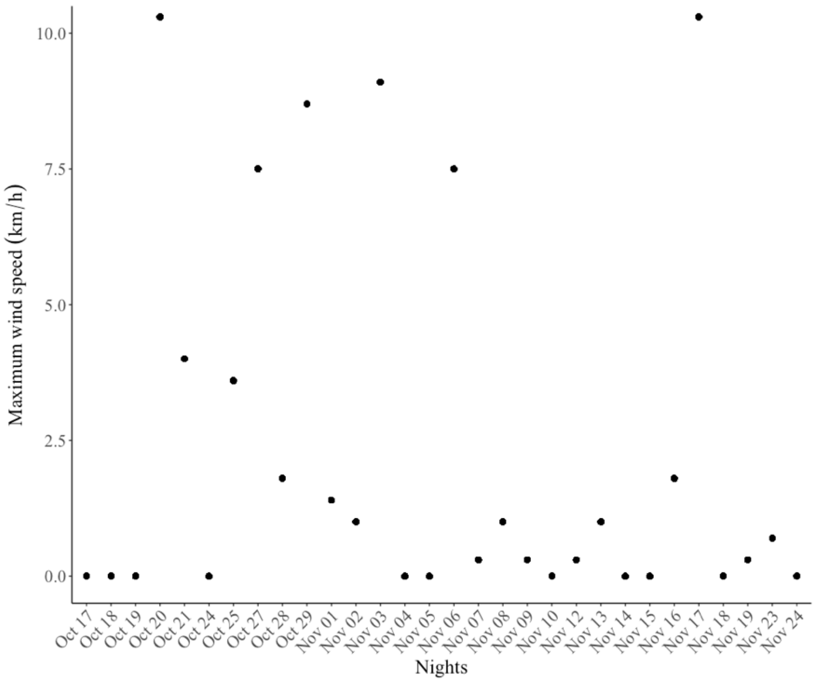

Supplementary Figure S6. Maximum wind speed (km/h) measured during the different twilight periods. Measurements were integrated along the entire observation period for each twilight period, which varied between 108 and 115 minutes (twilight duration).

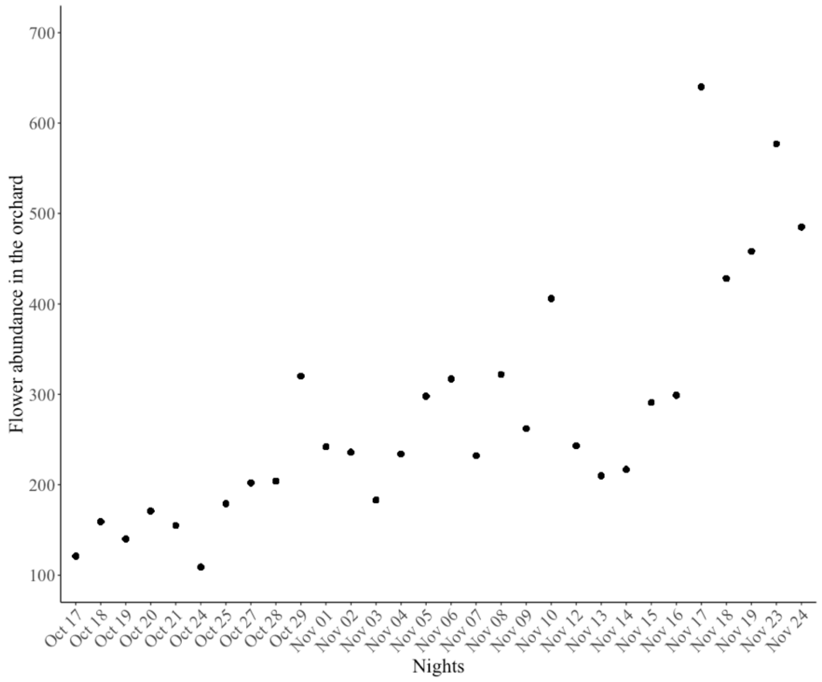

Supplementary Figure S7. Flower abundance in the cambuci orchard measured during the different twilight periods. Estimates were based on the total number of open flowers from 20 randomly selected cambuci trees.

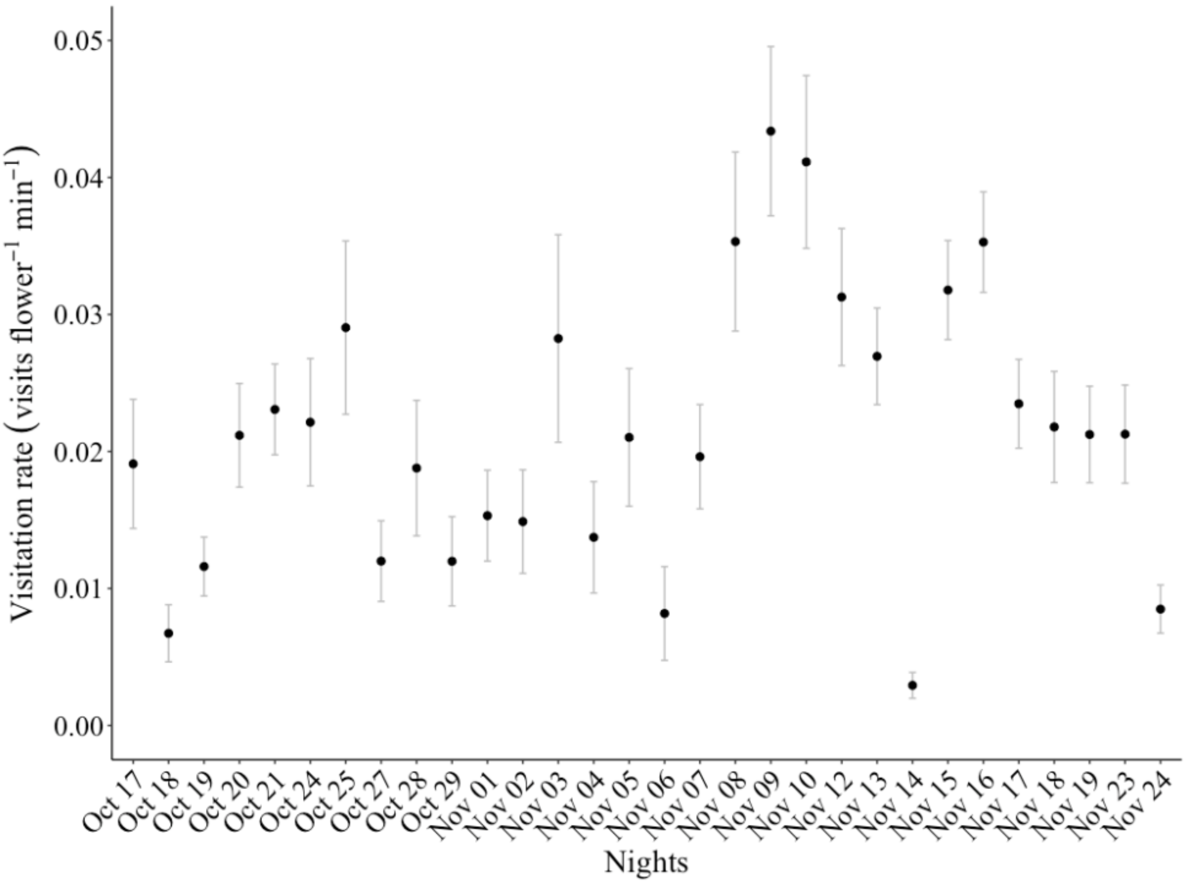

Supplementary Figure S8. Visitation rates (visits/flower/min) of nocturnal bees on cambuci flowers during the first half of the cambuci flowering season of 2017. *Dots* are average values and *bars* are standard errors of the mean ($r^2 = 0.03$; $y = 0.0164 + 0.0003x$; $p = 0.17$).

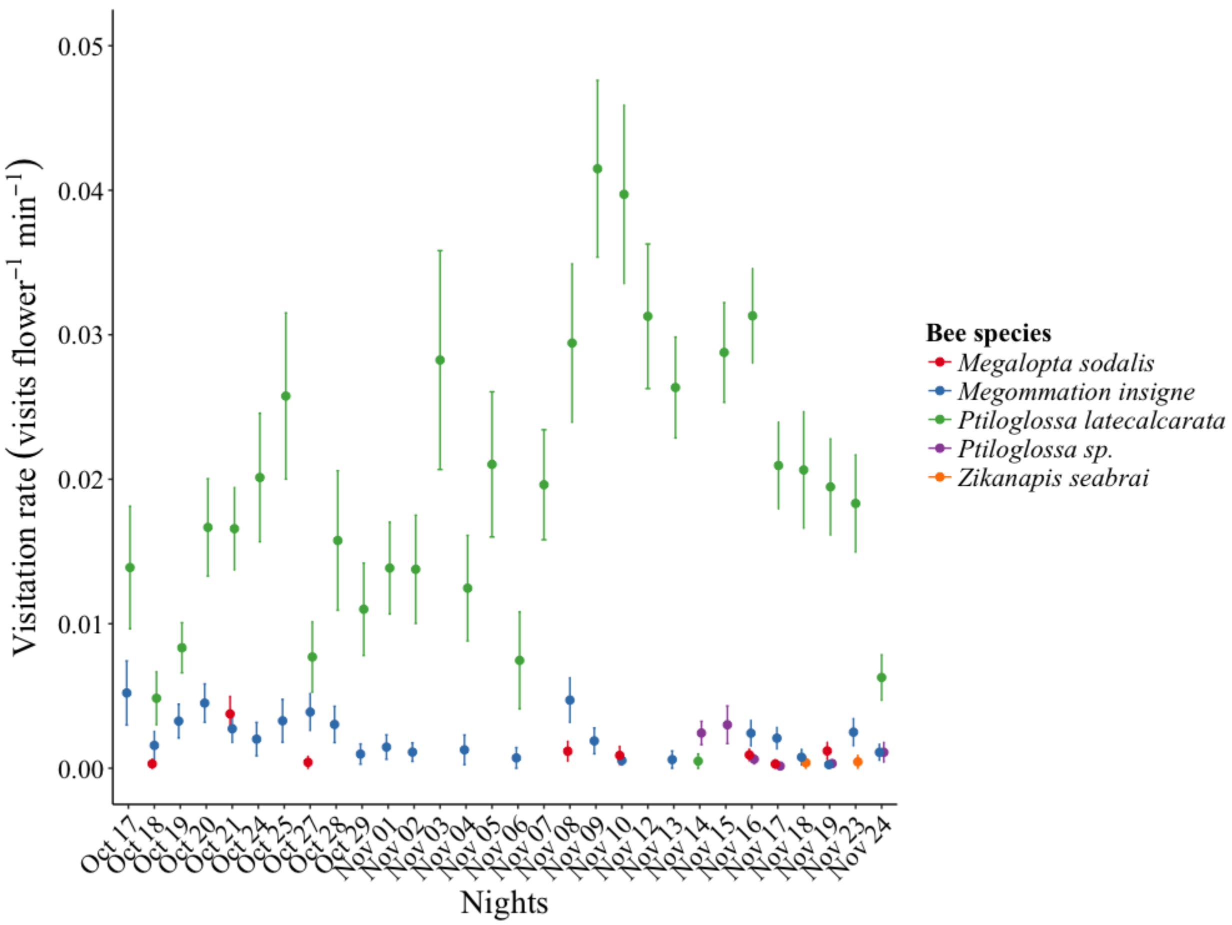

Supplementary Figure S9. Visitation rate (visits/flower/min) from five nocturnal bee species on cambuci flowers during the first half of the cambuci flowering season of 2017. *Dots* are average values and *bars* are standard errors of the mean.

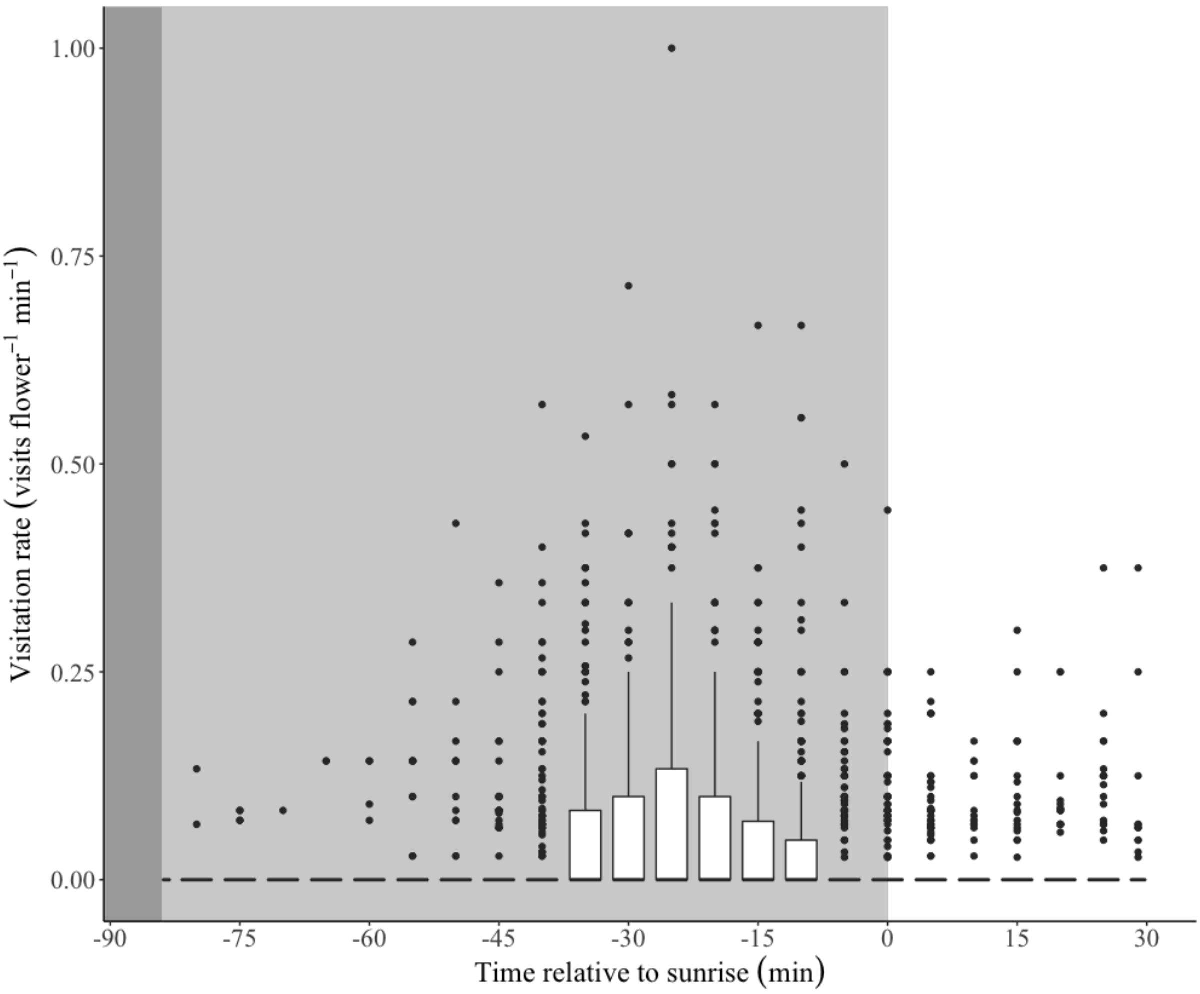

Supplementary Figure S10. The distribution of visitation rates (visits/flower/min) of nocturnal bees on cambuci flowers during twilight, aggregated in intervals of 5 min. *Boxes* delimit where 50% of the values are concentrated, *horizontal lines* indicate the median (all of them are zero), and *dots* represent outliers. Other plot conventions as in Fig. S1.

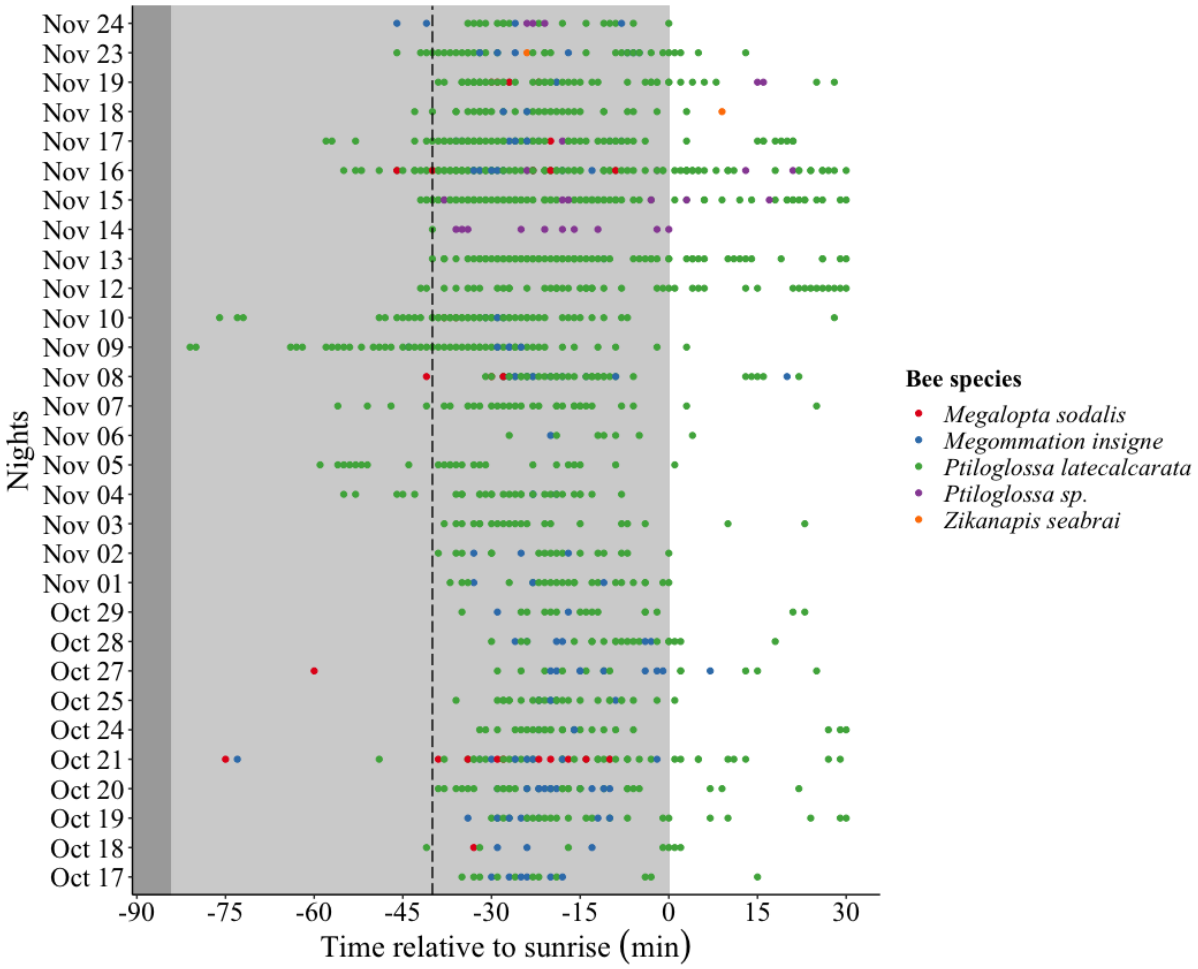

Supplementary Figure S11. Foraging activity durations of nocturnal bees on cambuci flowers measured during the different twilight periods and separated on the basis of species. Each *dot* represents the time at which a bee visited a cambuci flower. Other plot conventions as in Fig. S1.

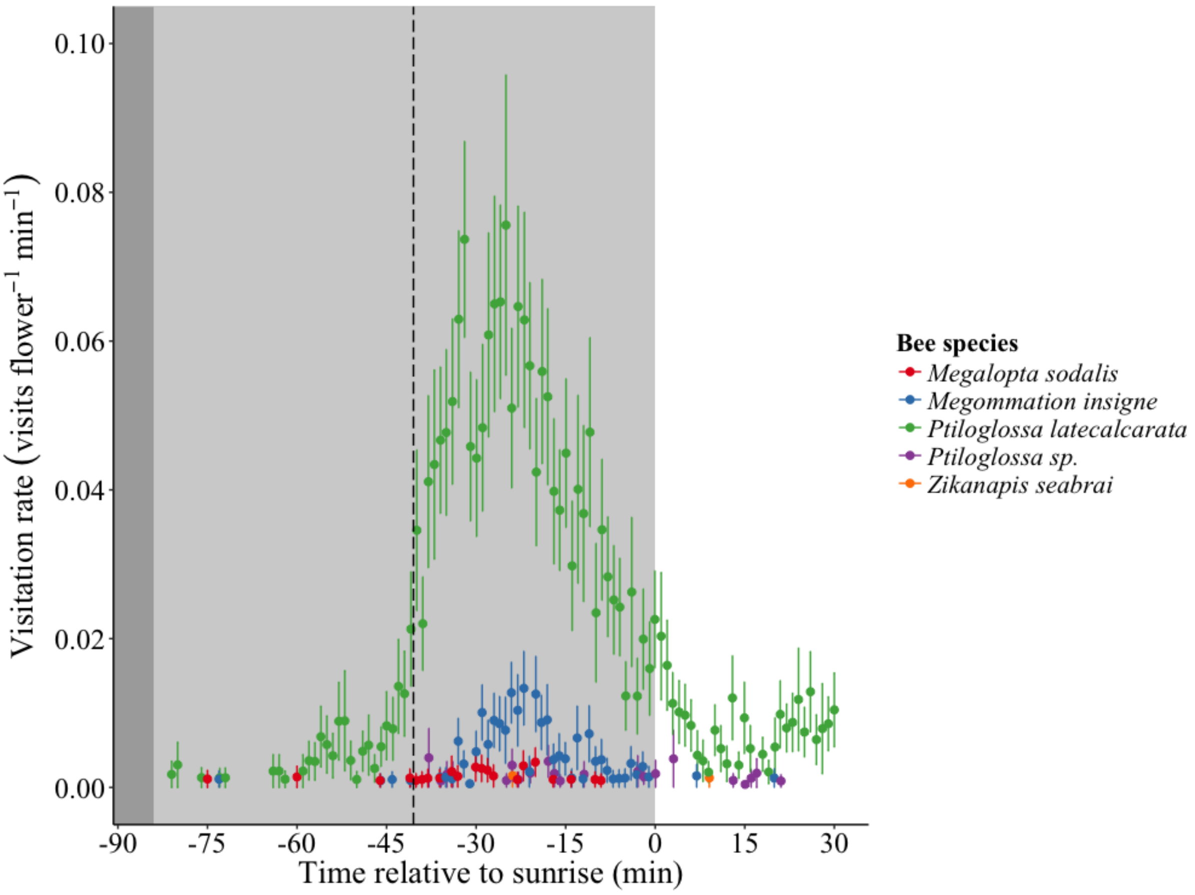

Supplementary Figure S12. Visitation rates (visits/flower/min) of five nocturnal bee species on cambuci flowers during twilight and directly after sunrise. *Dots* are average values and *bars* are standard errors of the mean. Other plot conventions as in Fig. S1.

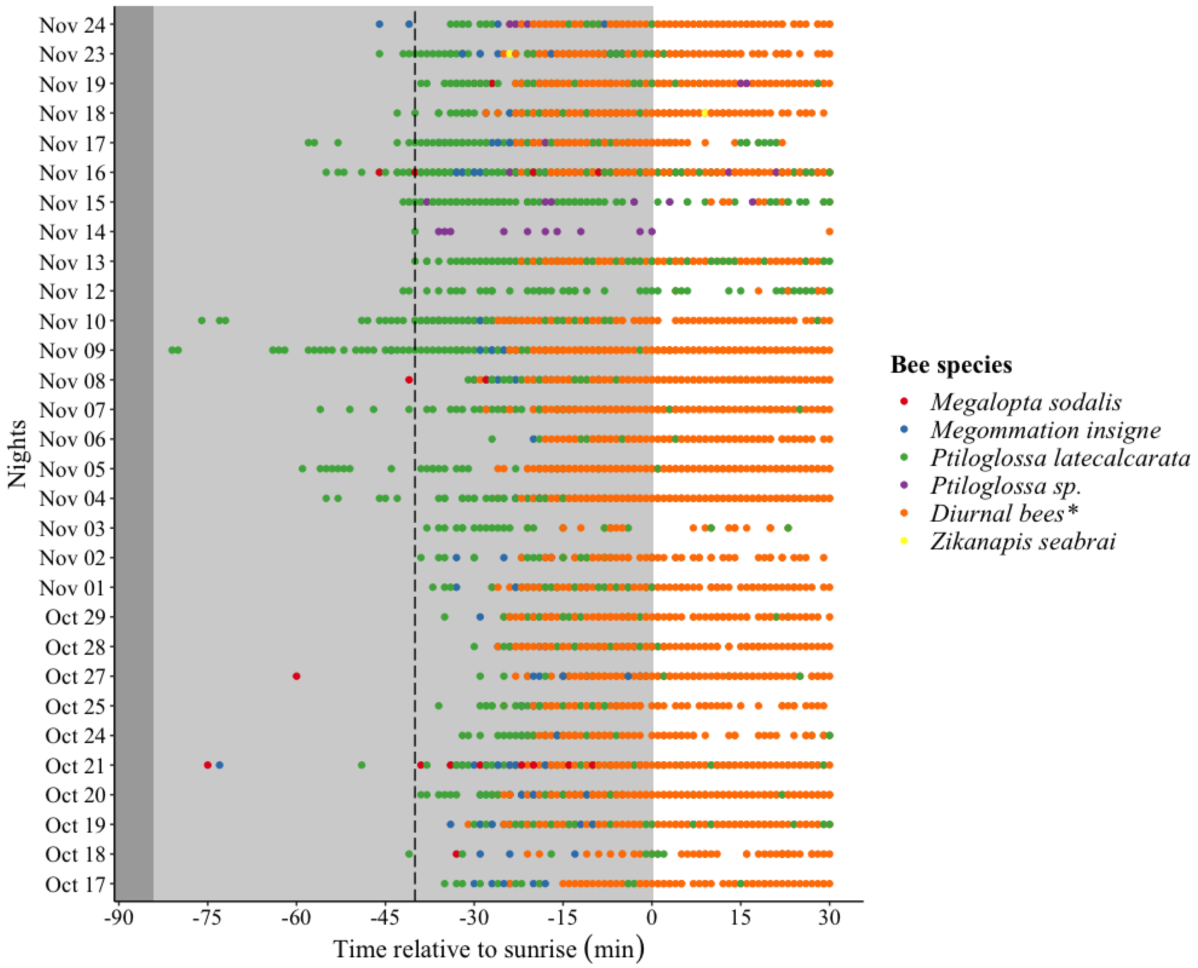

Supplementary Figure S13. Foraging activity durations of nocturnal and diurnal bees on cambuci flowers measured during the different twilight periods and separated on the basis of species. Each *dot* represents the time at which a bee visited a cambuci flower. * Diurnal bees included only social bees, mostly *Apis mellifera* (99.65% of the diurnal visits/flower/min). Other plot conventions as in Fig. S1.

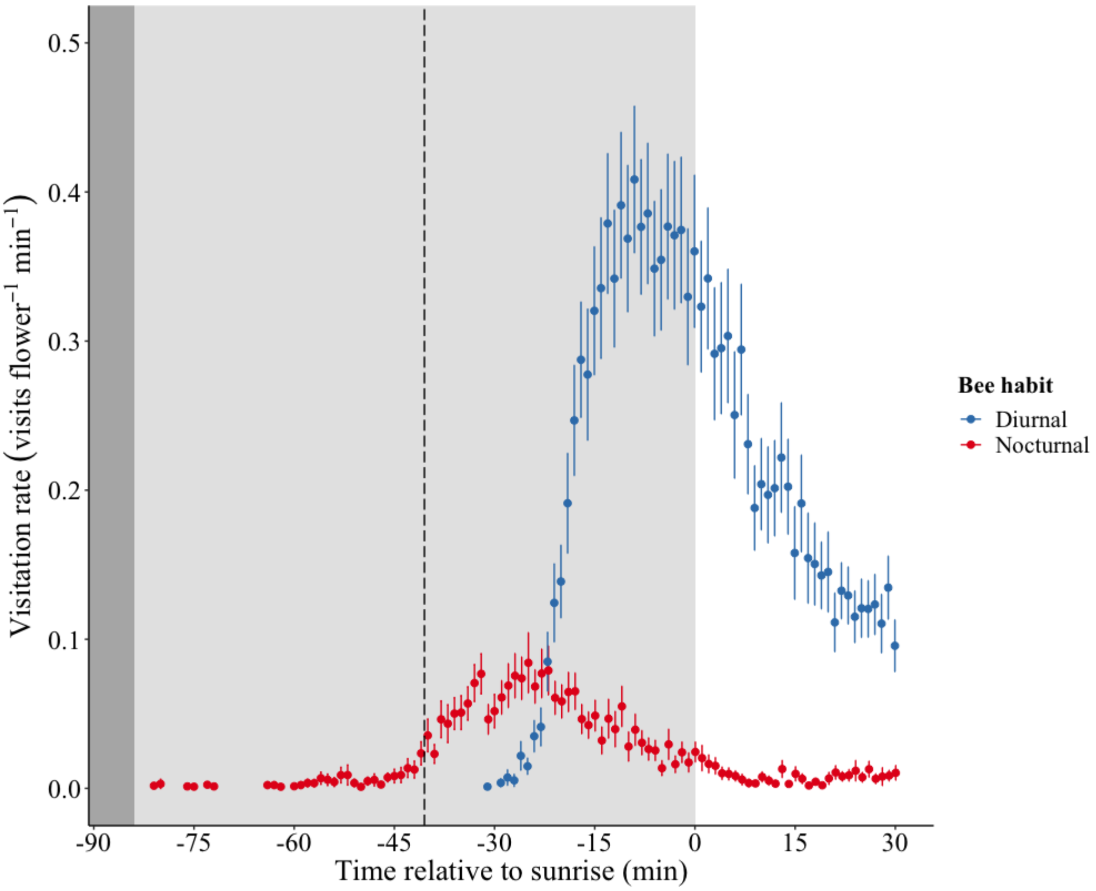

Supplementary Figure S14. Visitation rate (visits/flower/min) from nocturnal and diurnal bee species on cambuci flowers during twilight and directly after sunrise. *Dots* are average values and *bars* are standard errors of the mean. Diurnal bees included only social bees, mostly *Apis mellifera* (99.65% of the diurnal visits/flower/min). Other plot conventions as in Fig. S1.

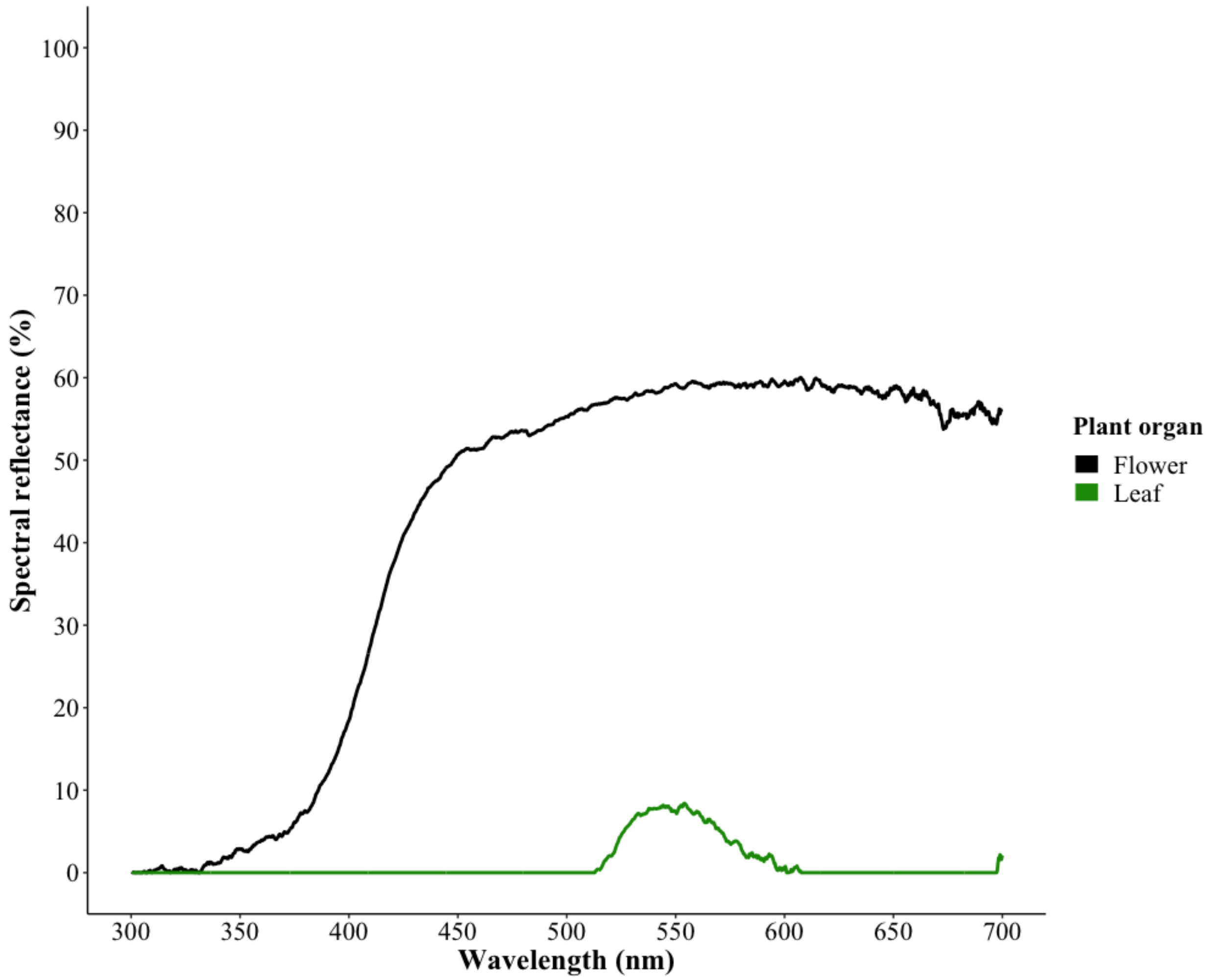

Supplementary Figure S15. Average spectral reflectance (%) of flowers and leaves of cambuci trees (*Campomanesia phaea*, Myrtaceae) from south-eastern Brazil. Data were taken from 11 flowers and 5 leaves from two individuals through spectrometer analysis (JAZ Spectrometer System, Ocean Optics, Inc., USA) at an angle of 45° to the measuring spot using a UV-VIS light source (400 µm, World Precision Instruments, Inc., USA).

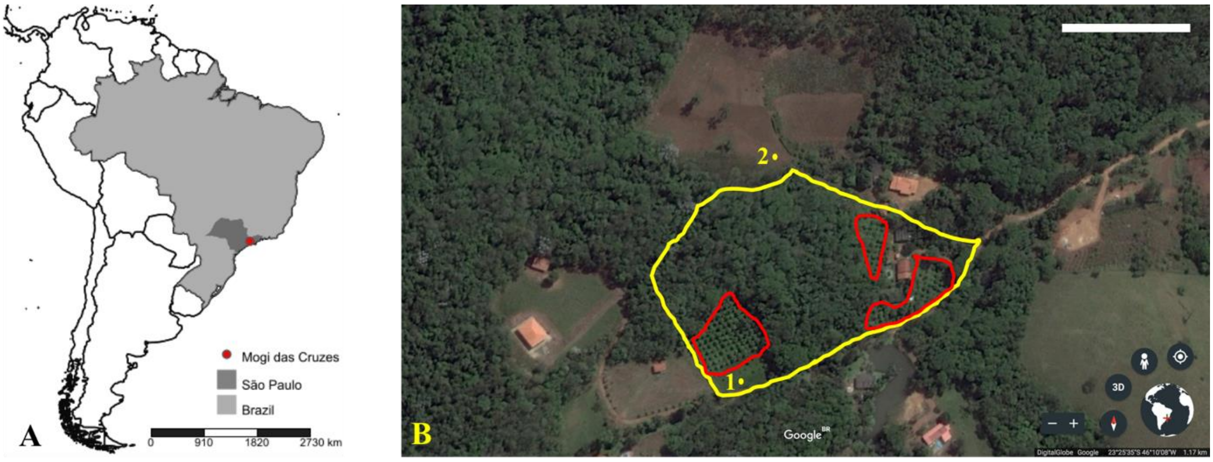

Supplementary Figure S16. Study area location. **A.** A map of South America indicating Mogi das Cruzes city, São Paulo State, and Brazil. **B.** A satellite picture of the cambuci orchard study site (Data source: Google, Maxar Technologies). The *yellow line* delimits cambuci farmland and *red lines* delimit cambuci orchards surrounded by secondary Atlantic forest fragments. *Dots* show the locations of the photometer (1), in an open area 10 m away from orchard, and the meteorological station (2), in an open area 70-100 m away from orchards. The *white scale bar* indicates 100 meters.

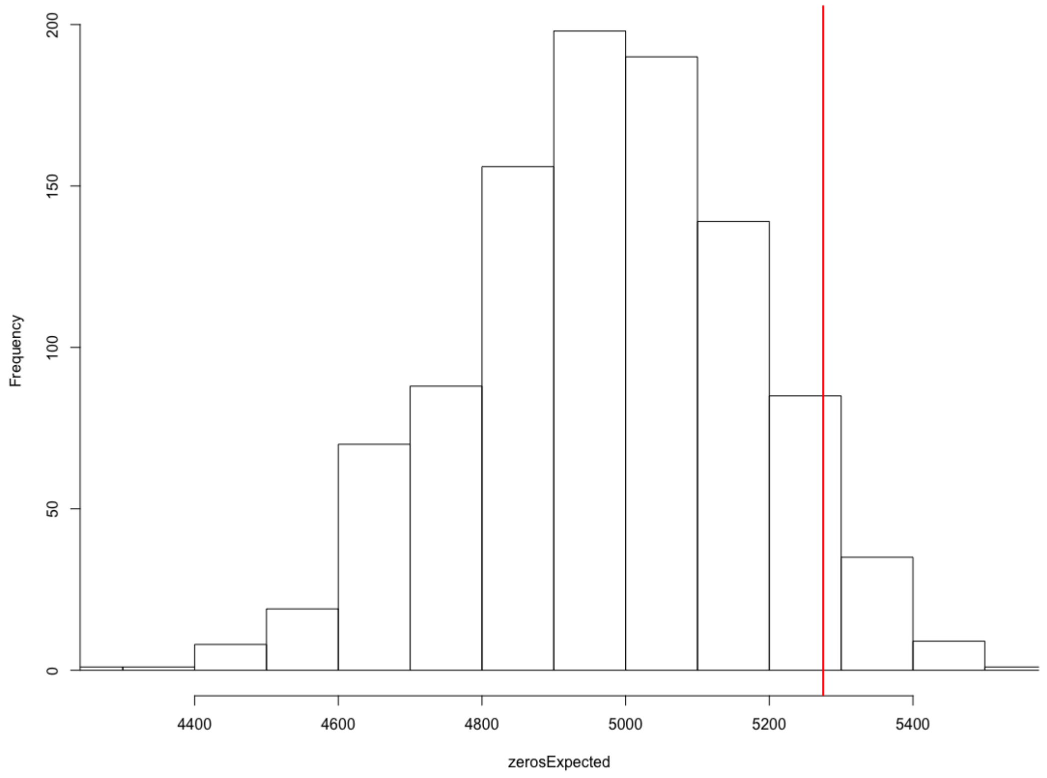

Supplementary Figure S17. Histogram of expected zeros for a zero-inflated dataset. According to this null distribution, our dataset cannot be considered as zero-inflated (ratioObsExp = 1.0603; p = 0.063).
